# The peroxisome: an update on mysteries 3.0

**DOI:** 10.1007/s00418-023-02259-5

**Published:** 2024-01-20

**Authors:** Rechal Kumar, Markus Islinger, Harley Worthy, Ruth Carmichael, Michael Schrader

**Affiliations:** 1 Faculty of Health and Life Sciences, Department of Biosciences, University of Exeter, Geoffrey Pope Building, Stocker Road, Exeter, EX4 4QD UK; 2 Institute of Neuroanatomy, Medical Faculty Mannheim, Mannheim Centre for Translational Neuroscience, University of Heidelberg, 68167 Mannheim, Germany

**Keywords:** Membrane contact sites, Motility, Organelle biogenesis, Organelle division, Organelle dynamics, Peroxin, Peroxisome, Protein import, STED microscopy

## Abstract

Peroxisomes are highly dynamic, oxidative organelles with key metabolic functions in cellular lipid metabolism, such as the β-oxidation of fatty acids and the synthesis of myelin sheath lipids, as well as the regulation of cellular redox balance. Loss of peroxisomal functions causes severe metabolic disorders in humans. Furthermore, peroxisomes also fulfil protective roles in pathogen and viral defence and immunity, highlighting their wider significance in human health and disease. This has sparked increasing interest in peroxisome biology and their physiological functions. This review presents an update and a continuation of three previous review articles addressing the unsolved mysteries of this remarkable organelle. We continue to highlight recent discoveries, advancements, and trends in peroxisome research, and address novel findings on the metabolic functions of peroxisomes, their biogenesis, protein import, membrane dynamics and division, as well as on peroxisome–organelle membrane contact sites and organelle cooperation. Furthermore, recent insights into peroxisome organisation through super-resolution microscopy are discussed. Finally, we address new roles for peroxisomes in immune and defence mechanisms and in human disorders, and for peroxisomal functions in different cell/tissue types, in particular their contribution to organ-specific pathologies.

## Introduction

Peroxisomes, which were discovered nearly 70 years ago (Rhodin 1954), are now recognised as key metabolic organelles with essential functions in cellular lipid metabolism (e.g., the β-oxidation of fatty acids and the synthesis of ether phospholipids, which contribute to myelin sheath formation) and the metabolism of reactive oxygen species (ROS), particularly hydrogen peroxide. Loss of peroxisomal function causes severe metabolic disorders in humans. Meanwhile, additional non-metabolic roles of peroxisomes have been revealed in cellular stress responses, regulation of cellular redox balance and healthy ageing, pathogen and antiviral defence, and as cellular signalling platforms. New findings also point to a role in the regulation of immune responses. These discoveries further highlight a “protective” role of these fascinating organelles, with their wider significance in human health encouraging exploration of their physiological roles and impact on globally important human diseases such as neurodegeneration, obesity, cancer, and age-related disorders.

In the tradition of our “mystery” series, we will continue to highlight recent discoveries, advancements, and trends in peroxisome research, which, we hope, will also provide non-experts and those who are not up to date with the current exciting developments with an overview of the field of peroxisome biology. This review represents an update and a continuation of three previous articles in our “mystery” series we published in *Histochemistry and Cell Biology* [the first and the third on the 50th and 60th anniversary of the journal in 2008 and 2018, respectively (Schrader and Fahimi 2008; Islinger et al. 2012, 2018)]. This review article is published on the occasion of the 65th anniversary of the journal in 2023. In our previous reviews, we addressed the role of peroxisomes in the brain, in neurological disorders, in the development of cancer, and in antiviral defence. To avoid repetition, we will refer to those articles where appropriate and to more specialised recent reviews on peroxisome biology.

## Super-resolution microscopy: insights into the mysteries of peroxisome organisation and morphology

Conventional fluorescence microscopy is a valuable tool for revealing many aspects of peroxisome biology; however, since a typical human peroxisome is roughly the same diameter as the wavelength of light, resolving detail at the sub-peroxisomal level using these techniques has proved elusive. While electron microscopy (EM) has been beneficially employed to understand peroxisomes on the ultrastructural level, it too possesses limitations as it cannot capture dynamic processes or be used to analyse large populations of cells and depends on epitope accessibility to localise proteins by immuno-EM. As a result, advances in super-resolution fluorescence microscopy, which is capable of overcoming limitations imposed by the diffraction limit of light, have recently accelerated our understanding of peroxisome organisation, morphology and interactions in an unprecedented level of detail (reviewed in Galiani et al. 2023).

**Fig. 1:**
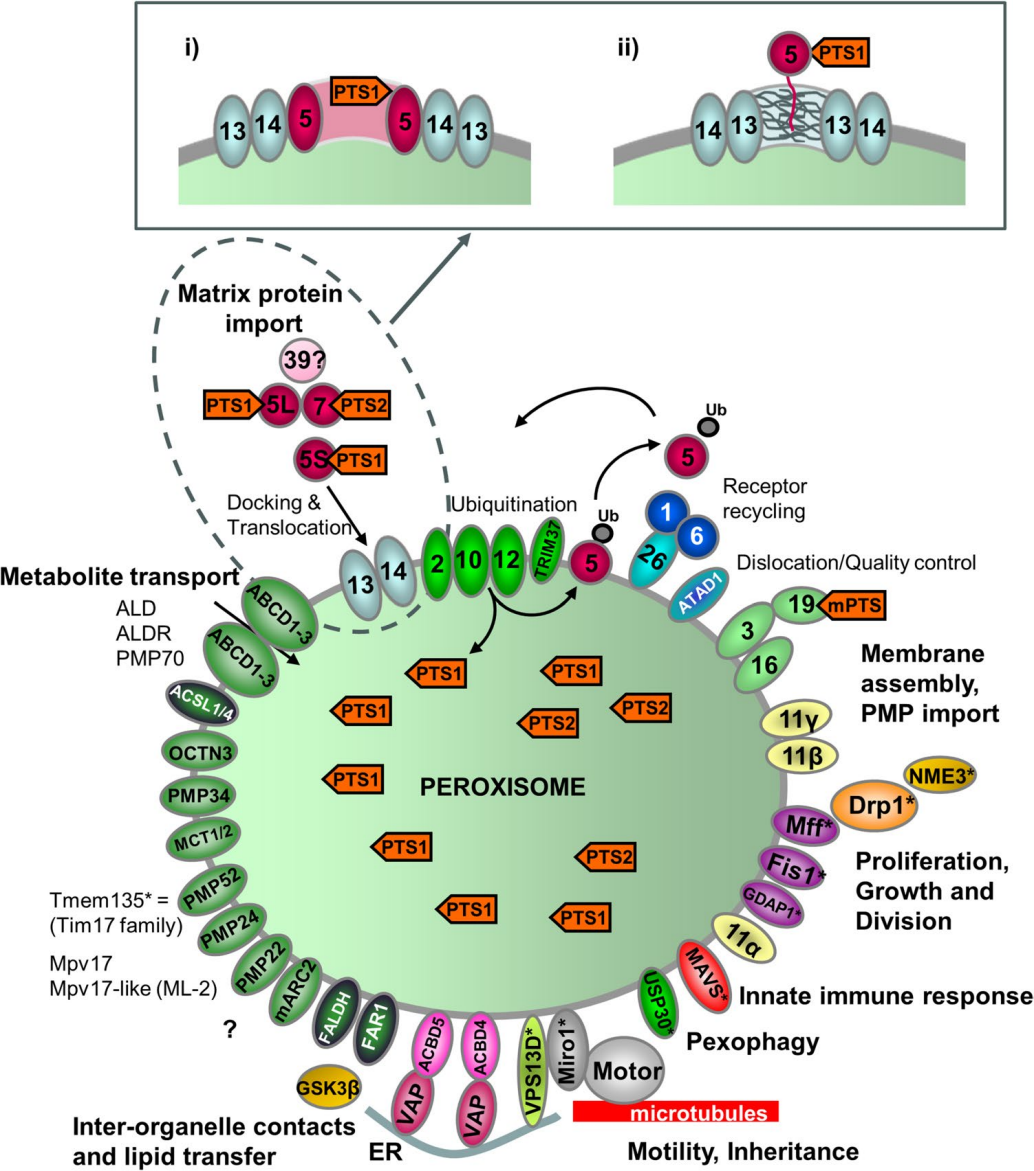
Schematic overview of the molecular machineries and proteins localised at the membranes of mammalian peroxisomes. Adapted from Schrader and Fahimi (2008). Related overviews for yeast and zebrafish peroxisomes can be found in Islinger et al. (2018) and Kamoshita et al. (2022). See text for further details. **Matrix protein import**: After synthesis on free ribosomes, cargo proteins containing the peroxisomal targeting signals PTS1 or PTS2 bind to the corresponding cytosolic receptors PEX5 or PEX7 and form receptor-cargo complexes. The PEX7–cargo complex requires accessory factors for import (PEX5L, a long isoform of PEX5, in mammals and plants, Pex18p and Pex21p in *Saccharomyces cerevisiae*, Pex20p in *Neurospora crassa*, *Yarrowia lipolytica*, and *Hansenula polymorpha*) (see Table 1). The newly discovered PEX39 may be involved in PTS2-mediated protein import. Import is achieved by a complex set of integral or peripheral PMPs that form the matrix protein import machinery, which mediates docking of the cargo-bound import receptor at the peroxisomal membrane, cargo translocation into the matrix of the organelle by a dynamic translocon, and export of the receptor back to the cytosol. Different models (top) have been proposed (see text for details): (i) PEX5 inserts into the peroxisomal membrane to form an oligomeric transient pore; (ii) PEX13 forms a stable pore, with YG repeats creating a hydrogel, with which PEX5 interacts. Recycling of the receptor involves its ubiquitination (Ub) and extraction from/through the membrane by an AAA-ATPase complex (PEX1, PEX6). PEX6 binds to PEX26 (Pex15p in yeast; see Table 1). **Membrane assembly and insertion of PMPs** (containing an mPTS) depends on PEX19, PEX3, and PEX16. PEX19 functions as a cycling receptor/chaperone, which binds the PMPs in the cytosol and interacts with PEX3 at the peroxisomal membrane. **Proliferation, growth and division**: PEX11α, PEX11β, and PEX11γ are involved in the regulation of peroxisome size and number (proliferation) in mammals. Mammalian PEX11β remodels the peroxisomal membrane and interacts with the membrane adaptors MFF and FIS1, which recruit the dynamin-like fission GTPase DRP1 (DRP3A in plants, Vps1p, Dnm1p in *S. cerevisiae*) to peroxisomes, which is activated by PEX11β. NME3 is supposed to supply GTP for DRP1-mediated fission. **Motility**: Mammalian peroxisomes move along microtubules, and MIRO1 serves as membrane adaptor for the microtubule-dependent motor proteins kinesin and dynein. **Tethering and lipid transfer**: ACBD5 and ACBD4 interact with ER-resident VAPA/B to mediate peroxisome-ER contacts in mammals. GSK3β regulates ACBD5-VAP interaction (Kors et al. 2022). VPS13D may be involved in phospholipid transfer from the ER to peroxisomes. ABCD1 is involved in peroxisome-LD contacts via M1 spastin. **Metabolite transport**: uptake of fatty acids is mediated by ABC transporter proteins (ABCD1-3 in mammals) (ALD, adrenoleukodystrophy protein; ALDR, ALD-related protein). **Other transporter and membrane proteins/enzymes**: OCTN3, organic cation/carnitine transporter 3; MCT1/2, monocarboxylate transporter 1/2; PMP52 (Tmem135) and PMP24 (PxmP4) belong to the Tim17 family (Žárský and Doležal 2016); members of the PMP22 family are Mpv17, Mpv17-like (ML-P); ACSL1/4, Acyl-CoA synthetase long-chain family member 1/4; Ant1, peroxisomal adenine nucleotide transporter 1; mARC2 (Mosc2), mitochondrial amidoxime reducing component 2; ATAD1/Msp1, ATPase family AAA (ATPase associated with various cellular activities) domain-containing protein 1; FALDH, fatty aldehyde dehydrogenase (Costello et al. 2017a); FAR1, fatty Acyl-CoA reductase 1 (ether lipid biosynthesis); GDAP1, ganglioside-induced differentiation-associated protein 1; MAVS, mitochondrial antiviral signalling protein; TRIM37, tripartite motif-containing protein 37; USP30, ubiquitin-specific protease 30 (Marcassa et al. 2018). Proteins with a dual localization to both peroxisomes and mitochondria are marked with an asterisk. *PEX* peroxin; *PMP* peroxisomal membrane protein

Stimulated emission depletion (STED) microscopy is a form of nanoscopy, meaning it is independent of light diffraction. The level of spatial resolution this affords can report on subtle morphological features of peroxisomes in both yeast and mammalian cells that would be invisible by other optical microscopy techniques (de Lange and Vlijm 2023), and as such has revealed a hitherto unappreciated heterogeneity in peroxisome size and shape (Galiani et al. 2016). Using STED microscopy to visualise peroxisomes in human fibroblasts, such as via dual-colour immunofluorescence of endogenous peroxisomal membrane (PMP70) (Fig. 1) and matrix (catalase or ACAA1) proteins, the peroxisome lumen and membrane can be clearly distinguished, with the signal corresponding to the matrix protein forming a central focus encircled by the membrane protein signal (Galiani et al. 2016; Soliman et al. 2018). Consequently, small but meaningful changes in peroxisomal membrane morphology and size can be discriminated and quantified. Quantitative analysis of peroxisomal membrane “ghosts” (empty peroxisomal membranes due to defects in matrix protein import) in fibroblasts from patients with Zellweger spectrum disorder (ZSD) by STED microscopy revealed that these are larger and less elongated than peroxisomes in control cells. Interestingly, cells with a higher level of residual matrix protein import (corresponding to a better patient prognosis) displayed larger and more complex “ghost” structures, highlighting a new sub-diffraction phenotype correlating with peroxisomal pathophysiology (Soliman et al. 2018).

Combining super-resolution microscopy with EM has unveiled the diversity of peroxisome morphology in even greater detail, and in the context of the crowded intracellular environment. Through correlating cryogenic super-resolution fluorescence microscopy of high-pressure frozen mammalian cells with focused ion beam scanning electron microscopy, peroxisomes labelled with a fluorescent matrix marker can be identified and reconstructed in three dimensions with EM-level resolution (Hoffman et al. 2020). This revealed that while smaller peroxisomes tend to be more spherical in shape, their morphology becomes more irregular as volume increases, adopting forms including flat plates, cups, and even hollow spheres. The consequence of this for peroxisomal function is currently unclear, but given that these irregularly shaped peroxisomes, with their increased surface area to volume ratio, can sometimes be observed in close proximity to other organelles, these morphologies may facilitate cooperative transfer of signals/metabolites at membrane contact sites.

**Fig. 2:**
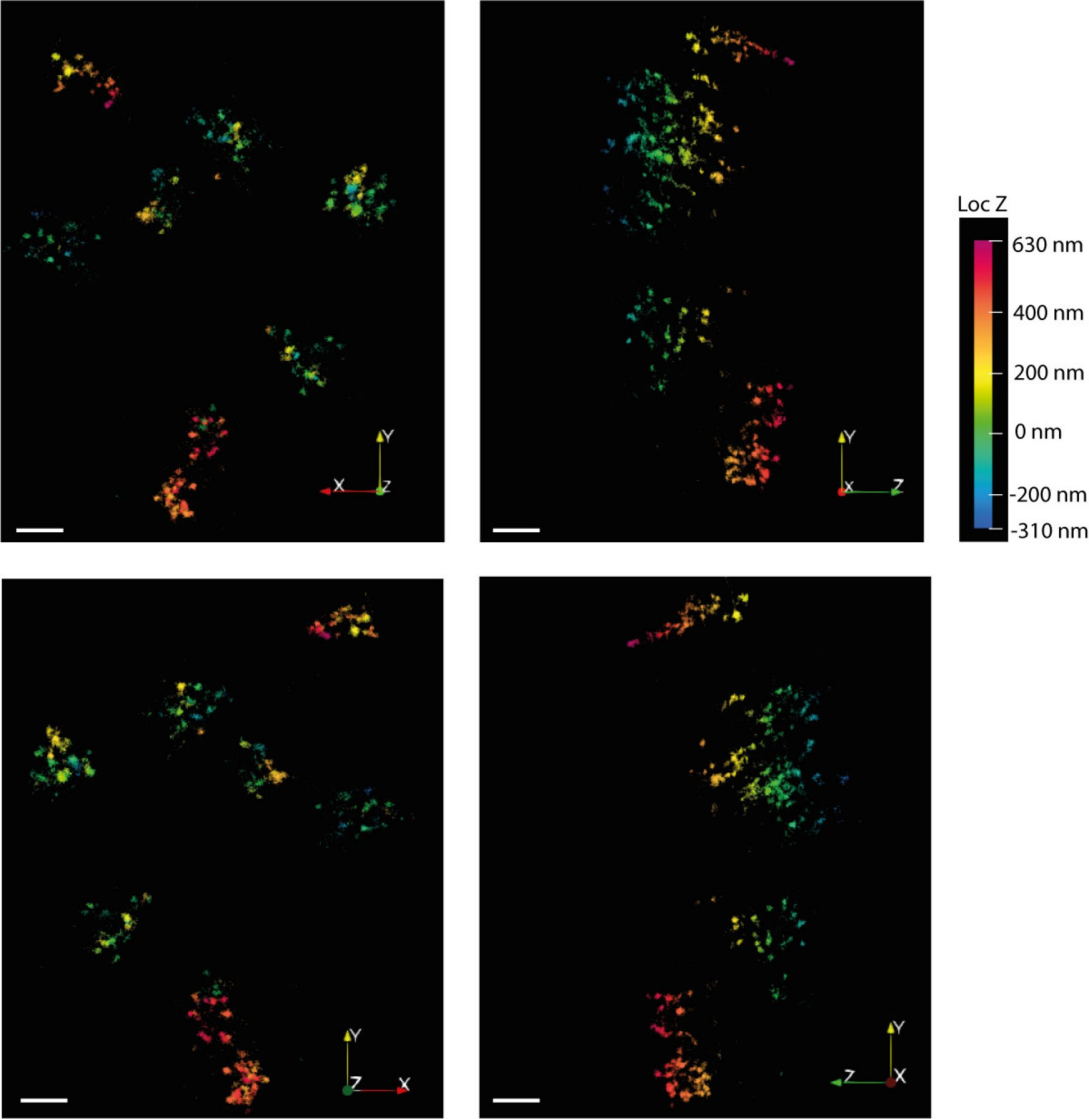
MINFLUX microscopy of peroxisomes. HEK293 cells expressing the peroxisomal membrane protein PEX26 with a SNAP Tag (SNAP-PEX26), which is labelled with Alexa 647 and imaged on a MINFLUX setup. Each cluster of puncta reflects subdomains of PEX26 in the membrane of a single peroxisome. The figure shows two-dimensional projections of a 3D MINFLUX recording from different angles, as indicated by the coordinates in the lower right corner. The colour code indicates the z position. Scale bar, 200 nm. Figure kindly supplied by K Reglinski/C Eggeling, University of Jena, Germany

Besides peroxisome morphology, super-resolution microscopy has also been exploited to reveal the sub-peroxisomal organisation of various proteins, within both the membrane and matrix. This has revealed that peroxisomal membrane proteins are not uniformly distributed through the membrane but show characteristic compartmentalisation into subdomains depending on the protein in question (e.g., PEX26, Fig. 2), which may be important for their distinct functions. In the majority of peroxisomes visualised by STED microscopy in human fibroblasts, endogenous PEX5 and PEX14, which are part of the matrix protein import machinery (Fig. 1), display a high level of colocalisation within small round structures at the membrane, consistent with their function together in the translocon (Galiani et al. 2016). However, PEX5 and PEX14 can sometimes also be seen in larger and more heterogeneous patches, which are characterised by lower colocalisation, and may represent peroxisomes with less active import, where PEX14 binding to PEX5 is outcompeted by other PEX14 binding partners including PEX19 and microtubules (Galiani et al. 2016). PEX11β, on the other hand, which is involved in peroxisomal membrane elongation and division (Fig. 1), is found in membrane-associated puncta that are much smaller and rounder compared to PEX5 or PEX14. These may represent regions of PEX11β oligomerisation and/or initiation points of membrane extension, but this has yet to be definitively shown (Galiani et al. 2016).

Compartmentalisation has also been observed for proteins within the peroxisomal matrix. While confocal microscopy of human fibroblasts expressing a GFP-tagged matrix protein (SCP2) shows typical homogeneous peroxisomal staining, applying STED microscopy reveals the signal to be heterogeneously and sometimes asymmetrically distributed within the bounds of the peroxisomal membrane (Galiani et al. 2016). This selective accumulation of proteins within subdomains of the peroxisomal matrix has been further interrogated in the fungus *Ustilago maydis* (Ast et al. 2022). Using the super-resolution technique of structured illumination microscopy (SIM), specific matrix enzymes in a subpopulation of peroxisomes are seen to localise to small foci within the lumen, which only partially colocalise with typical fluorescent matrix markers. The presence of the short peptide motif TIIV at the N-terminus of imported proteins is reportedly sufficient to recruit them to these foci within the matrix, likely representing the crystalline core structure, in both *U. maydis* and mammalian cells (Ast et al. 2022). The functional significance of this remains to be seen, but it may be a mechanism to generate different subpopulations of peroxisomes with unique protein content and thus specialised functions (Ast et al. 2022).

In more recent advances, STED microscopy has moved from providing predominantly descriptive data showing where peroxisomal proteins are precisely localised, to begin to reveal mechanistic details of their behaviour and dynamics. In combination with fluorescence correlation spectroscopy, STED microscopy has been exploited to reveal the cytosolic dynamics of PEX5, the receptor for cargo proteins containing peroxisomal targeting signal 1 (PTS1) sequences that are destined for matrix import (Galiani et al. 2022). Fluorescently labelled PEX5 freely co-diffuses with GFP-PTS1 cargo in three dimensions through the cytosol, but more slowly than would be expected for its molecular weight. This slow diffusion of PEX5 is not a result of the cargo binding itself, self-interaction, or interactions with the peroxisomal membrane, but instead is likely to result from a stable interaction with an as-yet unidentified cytosolic binding partner (Galiani et al. 2022).

Overall, super-resolution fluorescence microscopy, combined with the advanced image analysis techniques needed to interpret and quantify the outputs, is revealing there to be significant heterogeneity both within and between individual peroxisomes, in terms of morphology and protein distribution, and has begun to provide unexpected insights into peroxisomal protein dynamics. The forthcoming challenge for super-resolution microscopy is to further push the resolving power of these techniques to reveal even more detail, down to the single-molecule level. Proof-of-principle studies have demonstrated the capability of several super-resolution single-molecule localisation microscopy (SMLM) techniques, including dSTORM (Klein et al. 2023), fluorescence lifetime DNA-PAINT (Oleksiievets et al. 2022), and MINFLUX (Schmidt et al. 2021; Galiani et al. 2023) (Fig. 2), to visualise peroxisomal proteins. The future application of these SMLM methods to biological questions has great potential to provide the resolution needed to reveal aspects of peroxisomal biology that have previously been overlooked due to technical limitations.

## Mysterious functions: peroxisomal metabolism updated

Peroxisomes are key metabolic organelles with an oxidative metabolism. Their crucial role in cellular lipid metabolism, such as the α- and β-oxidation of fatty acids, the biosynthesis of ether phospholipids (e.g., myelin sheath lipids), the detoxification of glyoxylate, and the metabolism of reactive oxygen species, in particular H_2_O_2_, makes them indispensable for human health. Their metabolic pathways and associated pathophysiological roles in disease have been recently addressed in an excellent and comprehensive review (Wanders et al. 2023). Studies to identify the complete proteome of peroxisomes are ongoing. By performing a high-content screen on fluorescently tagged yeast proteins, Yifrach et al. (2022) identified 33 additional peroxisomal proteins, thus increasing the inventory of the yeast peroxisomal proteome to 115 proteins. Functional mapping of targeting dependencies for matrix proteins revealed multiple non-canonical Pex5 substrates. Peroxisomal targeting of GID (glucose-induced degradation deficient) complex subunits was also uncovered, which places peroxisomes as regulators of gluconeogenesis. Moreover, two additional potential peroxisomal lipases (Ykl050c [Lpx2], Fsh3) were identified. They may play a role in membrane remodelling and/or the degradation of intraluminal vesicles as a source of free fatty acids. Interestingly, intraluminal vesicles with roles in fatty acid catabolism and protein compartmentalisation have been observed in plants (Wright and Bartel 2020) and in yeast (Thoms et al. 2008).

The maintenance of NAD(H) homeostasis in peroxisomes is crucial for peroxisomal fatty acid β-oxidation, but its regulation has been mysterious. Using peroxisome-targeted NADH biosensors, Chornyi et al. now show that the NAD+/NADH ratio in the cytosol and peroxisomes is closely connected (Chornyi et al. 2023a). This crosstalk is mediated by intraperoxisomal lactate and malate dehydrogenases, which are generated via translational stop codon readthrough of the LDHB and MDH1 mRNAs (Schueren et al. 2014; Hofhuis et al. 2016). Apparently, two independent redox shuttle systems are exploited by human peroxisomes to regulate peroxisomal NAD+/NADH homeostasis.

Further insight has also been provided on the maintenance of the peroxisomal redox equilibrium (Costa et al. 2023; Ferreira et al. 2023). Although peroxisomal oxidases generate large amounts of H_2_O_2_, cysteine residues of intraperoxisomal proteins are maintained in a reduced state. Glutathione S-transferase 1 kappa (GSTK1) is the only human peroxisomal glutathione-consuming enzyme identified so far. Loss of GSTK1 has now been observed to leave the basal intraperoxisomal redox state unaltered but to significantly extend the recovery period of the peroxisomal glutathione redox sensor po-roGFP2 upon treatment of the cells with thiol-specific oxidants. These findings demonstrate that GSTK1 contains GSH-dependent disulfide bond oxidoreductase activity (Costa et al. 2023). Furthermore, by establishing a cell-free in vitro system to provide isolated rat liver peroxisomes with a glutathione redox sensor, it has been demonstrated that the peroxisomal membrane is permeable to both reduced and oxidised glutathione, and that the intraperoxisomal and cytosolic pools of glutathione are redox linked (Ferreira et al. 2023). The findings suggest that glutathione plays a key role in maintaining intraperoxisomal redox homeostasis.

Proper functioning of peroxisomes in metabolism requires the concerted interaction with other subcellular organelles including the ER, mitochondria, LDs, lysosomes, and the cytosol. The metabolic cooperation between peroxisomes and other organelles involves the formation of membrane contact sites (MCSs) by tether proteins, which allow efficient transfer of metabolites (Silva et al. 2020) (see “Progress towards understanding the mysteries of peroxisome-organelle membrane contact sites”). A striking example of peroxisome-ER metabolic cooperation is the de novo biosynthesis of ether phospholipids. The first steps occur in peroxisomes and are mediated by the peroxisomal matrix enzymes glyceronephosphate O-acyltransferase (GNPAT/DHAPAT) and alkylglycerone phosphate synthase (AGPS/ADHAPS). The latter is the only enzyme able to generate the characteristic ether bond. Furthermore, FAR1 and FAR2, two fatty acyl-CoA reductases attached to the cytosolic side of the peroxisomal membrane, are involved (Fig. 1). The remaining steps in ether lipid synthesis take place at the ER. Peroxisomal GNPAT, which mediates the first step in de novo ether lipid synthesis, has a strict substrate specificity and reacts only with long-chain acyl-CoAs (Ofman and Wanders 1994). However, the origin of these long-chain acyl-CoAs remained a mystery, and it was unclear whether they were produced inside the peroxisomes or imported from the cytosol, and which enzymes and transporters were involved. Chornyi and co-workers have now developed a sensitive LC–MS-based method to investigate de novo ether lipid synthesis in a variety of HeLa knockout cell lines (Chornyi et al. 2023b). They revealed that the long-chain acyl-CoA esters required for the first step of the ether lipid synthesis are imported into human peroxisomes from the cytosol by the peroxisomal ABCD transporters, in particular by ABCD3 (Fig. 1) but are also produced through chain shortening of very-long-chain fatty acids inside peroxisomes via β-oxidation. These findings highlight the intimate connection between peroxisomal β-oxidation and ether lipid synthesis and the crucial role of the peroxisomal ABCD transporters in this process (Chornyi et al. 2023b).

Peroxisomes also interact and cooperate with lipid droplets (LDs) and mitochondria in fatty acid metabolism (Silva et al. 2020; Wanders et al. 2023). Fatty acids, which are stored in LDs, are mobilised under lipolytic conditions, and are then routed to peroxisomes and mitochondria for β-oxidation with subsequent ATP production in mitochondria. In yeast, fatty acid β-oxidation is solely peroxisomal, whereas in animals and some other fungi, both peroxisomes and mitochondria possess a β-oxidation pathway. However, peroxisomes in animals/mammals can only chain-shorten fatty acids to a length of C8 and need to route their β-oxidation products to mitochondria for full oxidation and ATP production. It remains a mystery how membrane contacts between these organelles are organised and regulated, and which proteins are involved in fatty acid transfer between these organelles. Recent work by Enkler et al. has now revealed that the GTPase Arf1 coordinates the transfer of free fatty acids from LDs to peroxisomes, and shortened acyl-CoAs from peroxisomes to mitochondria (Enkler et al. 2023). In yeast, expression of the gain of function Arf1-11 mutant results in a reduction of fatty acid transporters Pax1 and Pax2, and the acyl-CoA oxidase Pox1 at yeast peroxisomes, leading to a reduction in β-oxidation but not to a reduced rate of peroxisome biogenesis. In parallel, the import of acetyl-CoA into mitochondria was shown to be inhibited in both yeast and mammalian cells. The authors propose that Arf1, which localises to contact sites between these organelles, regulates mitochondrial function by controlling the flow of fatty acids and metabolites from LDs to peroxisomes/mitochondria in yeast and mammalian cells (Enkler et al. 2023) (see “Progress towards understanding the mysteries of peroxisome-organelle membrane contact sites”).

Another important function of peroxisomes is the β-oxidation of bile acid intermediates in the liver. A very recent study has now revealed the first evidence for a core function of the SUMO and ubiquitin E3 ligase MAPL as a key regulator of bile acid metabolism (Goyon et al. 2023). MAPL localises to mitochondria and peroxisomes. The peroxisomal bile acid transporter ABCD3/PMP70 has been identified as SUMO substrate of MAPL, with SUMOylation repressing the transporter. Loss of MAPL resulted in increased bile acid production coupled with defective regulatory feedback in liver. Furthermore, mice lacking MAPL present a series of consequential phenotypes, from production of the stress hormone FGF21 and insulin sensitivity to the generation of hepatocellular carcinoma. The study provides insight into the post-translational regulation of bile acid metabolism within the liver and the central role for peroxisomal SUMOylation in metabolic homeostasis.

Whether peroxisome-organelle communication involves calcium signalling, and whether peroxisomes are sites for calcium handling and exchange has remained a mystery. Sargsyan et al. developed several peroxisomal calcium sensors and showed that peroxisomes in HeLa cells and cardiomyocytes (see “Unravelling the mysteries of peroxisomes in discrete places”) can take up calcium upon cytosolic calcium increase after both ER calcium-store depletion and calcium entry to the cells across the plasma membrane (Sargsyan et al. 2021, 2022). The findings indicate that under specific conditions, peroxisomes may function as additional calcium buffers and take up excessive calcium to protect the cells. If calcium also regulates peroxisomal metabolic processes remains to be elucidated.

## Some mysteries about peroxisomal matrix protein import disclosed?

### Novel peroxins

Peroxisome biogenesis depends on peroxins (PEX proteins), which are essential for peroxisome formation and include proteins of the import machineries for matrix and membrane proteins as well as proteins involved in peroxisome multiplication/proliferation (Fig. 1). The human genome encodes for 14 peroxins, with defects in those linked to severe disorders of the Zellweger spectrum (Braverman et al. 2014) (see “Mysterious disorders: the loss of peroxisomal functions”). However, additional peroxins have been identified in other organisms, in particular in yeast. Since our last review in 2018, the number of identified peroxins has further increased to 39 (Table 1). We already reported on the discovery of Pex9, a Pex5-like yeast peroxisomal targeting receptor, which is involved in the targeting of a subset of PTS1-containing matrix proteins during growth in oleate (Effelsberg et al. 2016; Yifrach et al. 2016). The peroxisomal membrane protein Pex35 is a regulator of peroxisome abundance in yeast (Yofe et al. 2017). Pex36 is a functional homolog of mammalian PEX16 which is involved in the ER-to-peroxisome traffic of PMPs (Farré et al. 2017). Recently, a new peroxin, *Hansenula polymorpha* Pex37, a membrane protein and member of the Pxmp2-related protein family, has been reported to be required for peroxisome fission and segregation (Singh et al. 2019; Platta and Erdmann 2019). A potential novel peroxin, PEX38, has been identified in *Trypanosoma brucei* as a binding partner of PEX19. It is supposed to act as a co-chaperone assisting PEX19 in PMP targeting (R. Erdmann, personal communication) (Table 1). The identification of a phylogenetically conserved novel peroxin, PEX39, with a specific role in the PTS2-mediated protein import pathway has very recently been reported (Pedrosa et al. 2023b). The yeast ortholog partially localises to peroxisomes, interacts with Pex18 and, when deleted, results in a growth defect under peroxisome-proliferating conditions and a cytosolic mislocalisation of PTS2 cargo proteins. The human ortholog interacts with a trimeric PEX5/PEX7/PTS2 complex and blocks the association of PEX7 with peroxisomes and the peroxisomal import of pre-thiolase, a PTS2 protein, in an in vitro import assay (Fig. 1; Table 1).

**Table 1:** Overview of identified peroxins across organisms/species

Peroxin	Known domains	Role	Molecular function	Distribution
PEX1	AAA-domain	Matrix protein import	ATPase which binds PEX6 and is involved in the dislocation of the PTS-receptors	Eukaryotes
PEX2	RING-domain	Matrix protein import	Forms the ubiquitin E3 ligase RING-complex together with PEX10 and PEX12 and is involved in the ubiquitination of the PTS-receptors	Eukaryotes
PEX3		PMP-targeting	Membrane anchor for PEX19; involved in de novo formation	Eukaryotes
PEX4		Matrix protein import	Ubiquitin-conjugating E2 enzyme which participates in monoubiquitination of PTS-receptors	Eukaryotes (except metazoa)
PEX5	WxxxF-motifs; TPR-domains, ubiquitinated	Matrix protein import	Receptor for the PTS1-signal; required for the PTS1-dependent matrix protein import; PEX5L isoform required for PTS2 import in mammals/plants	Eukaryotes
PEX6	AAA-domain	Matrix protein import	ATPase which binds PEX1 and is involved in the dislocation of the PTS-receptors	Eukaryotes
PEX7	WD40 domain	Matrix protein import	Receptor for the PTS2-signal; required for the PTS2-dependent matrix protein import; forms complex with PTS2-co-receptors (PEX5L in mammals/plants; PEX18/PEX20/PEX21 in yeast)	Eukaryotes
PEX8	Coiled-coil domain, leu-zipper	Matrix protein import	Peripheral intraperoxisomal membrane protein; bridges docking- and RING-complex; possibly involved in cargo disassembly	Fungi
PEX9	WxxxF motif; TPR-domains	Matrix protein import	Condition-specific PTS1 receptor (arising from PEX5 duplication)	*Saccharomyces cerevisiae*
PEX10	RING-domain	Matrix protein import	Forms the ubiquitin E3 ligase RING-complex together with PEX2 and PEX12 and is involved in the ubiquitination of the PTS-receptors	Eukaryotes
PEX11*	Amphipathic helices	Proliferation	Peroxisome biogenesis; elongation of peroxisomes	Eukaryotes
PEX12	RING-domain	Matrix protein import	Forms the ubiquitin E3 ligase RING-complex together with PEX2 and PEX10 and is involved in the ubiquitination of the PTS-receptors	Eukaryotes
PEX13	SH3-domain, FY repeats	Matrix protein import	Integral membrane protein required for the docking of receptor/cargo complexes; forms docking complex with PEX14 and PEX17	Eukaryotes
PEX14	PXXP-domain	Matrix protein import	Membrane protein required for the docking of receptor/cargo complexes; forms docking complex with PEX13 and PEX17	Eukaryotes
PEX15		Matrix protein import	Phosphorylated tail-anchored PMP that is involved in the recruitment of yeast PEX6 to the peroxisomal membrane	*Saccharomyces cerevisiae*
PEX16		PMP-targeting; proliferation	Membrane protein that interacts with PEX3 and PEX9; involved in de novo biogenesis	Eukaryotes
PEX17		Matrix protein import	Membrane-associated protein that forms the docking complex with PEX14 and PEX13	Fungi
PEX18		Matrix protein import	PTS2-co-receptor; interacts with PEX7 (arising from PEX20 duplication); partially redundant with PEX21	*Saccharomyces cerevisiae*
PEX19	CAAX-box; farnesylated	PMP-targeting	PMP receptor and chaperone; involved in de novo formation	Eukaryotes
PEX20	WxxxF-motifs; ubiquitinated	Matrix protein import	Involved in PTS2-dependent protein import, mostly as co-receptor of PEX7 (in most fungi, e.g., *Pichia pastoris*)	Fungi
PEX21	WxxxF-motifs; ubiquitinated(?)	Matrix protein import	Interacts with PTS2-receptor PEX7 (arising from PEX20 duplication)	*Saccharomyces cerevisiae*
PEX22		Matrix protein import	Membrane protein required for the recruitment of PEX4 to the peroxisomal membrane	Fungi, plants, and protists
PEX23**	DysF	Proliferation	Growth regulation	Fungi
PEX24***	DysF	Proliferation	Separation of peroxisomes (similarity to PEX28)	Fungi
PEX25		Proliferation	Peroxisome biogenesis; elongation of peroxisomes	Fungi
PEX26		Matrix protein import	Tail-anchored PMP that is involved in the recruitment of PEX6 to peroxisomal membranes	Metazoa and fungi
PEX27*		Proliferation	Elongation of peroxisomes	*Saccharomyces cerevisiae*
PEX28***	DysF	Proliferation	Separation of peroxisomes (similarity to PEX24)	Fungi
PEX29***	DysF	Proliferation	Separation of peroxisomes	Fungi
PEX30**	DysF	Proliferation	Growth regulation (arising from duplication of PEX23)	*Saccharomyces cerevisiae*
PEX31**	DysF	Proliferation	Growth regulation (arising from duplication of PEX23)	*Saccharomyces cerevisiae*
PEX32**	DysF	Proliferation	Growth regulation	Fungi
PEX33		Matrix protein import	Part of the docking complex in filamentous fungi, e.g., *N. crassa*	Fungi
PEX34*		Proliferation	Membrane protein implicated in de novo biogenesis	Fungi
PEX35		Proliferation	Regulator of peroxisome abundance in budding yeast; associates with PEX11 family proteins	Saccharomycetaceae
PEX36^(*)^		PMP-targeting	Functional homolog of PEX16; implicated in de novo biogenesis	Fungi
PEX37		Proliferation	Pxmp2-related membrane protein involved in peroxisome fission and separation	Fungi
PEX38 (proposed)		PMP-targeting	Co-chaperone assisting PEX19 in PMP targeting	*Trypanosoma brucei*
PEX39 (proposed)		Matrix protein import	Yeast ortholog involved in peroxisome proliferation. Human ortholog blocks import of pre-thiolase in vitro	Eukaryotes

Adapted from Platta and Erdmann (2007); Hasan et al. (2013); and Jansen et al. (2021). *PEX11 family; **PEX23 subfamily, ***PEX24 subfamily. See text for details

Besides the peroxins, which are usually peroxisomal membrane proteins or can associate with the peroxisomal membrane, the peroxisomal membrane contains a number of transporters for metabolite and co-factor exchange (Fig. 1). Our current knowledge about these peroxisomal transporters has recently been summarised in an excellent review article (Chornyi et al. 2020). Evidence for a complementary system for the uptake of ATP in peroxisomes has been provided through studies in the yeast *S. cerevisiae*. Intra-peroxisomal ATP levels appear to be maintained by different peroxisomal membrane proteins. Ant1p catalyses the exchange of ATP for AMP or ADP; the Pxa1p/Pxa2p ABC transporter protein complex mediates both uni-directional acyl-CoA and ATP uptake; and Aac2p, which dually localises to peroxisomes and mitochondria, catalyses ATP/ADP exchange (van Roermund et al. 2022). Furthermore, evidence from in vitro studies revealed that the mammalian peroxisomal membrane is permeable to both reduced and oxidised glutathione and that the intra-peroxisomal and cytosolic pools of glutathione are redox linked (see “Mysterious functions: peroxisomal metabolism updated”). Glutathione appears to play a key role in protecting cysteine residues of peroxisomal matrix proteins from oxidation by H_2_O_2_, which is generated by several peroxisomal oxidases (Ferreira et al. 2023).

### The mysterious pore

A remarkable and unique property of the peroxisomal matrix protein import machinery is its ability to transport folded proteins and even assembled protein complexes into peroxisomes (Walton et al. 1995). This involves binding of newly synthesised peroxisomal matrix proteins by the cytosolic import receptor PEX5 through the receptor’s tetratricopeptide repeat (TPR) domain (Fig. 1). Most of the mammalian cargo proteins contain a PTS1 at the very C-terminus, explaining why the proteins need to be imported post-translationally. The “transient pore” model postulates that the PEX5-cargo complex inserts into the peroxisomal membrane and initiates the formation of a large pore for cargo translation, which is however short-lived in nature (Erdmann and Schliebs 2005; Meinecke et al. 2010; Walter and Erdmann 2019; Blum et al. 2023; Rudowitz and Erdmann 2023) (Fig. 1 top). How these processes occur is still a mystery, and a pore structure has not yet been visualised.

Other findings do not support a role for PEX5 as an integral component of the translocation channel in the peroxisomal membrane (Dias et al. 2017; Skowyra and Rapoport 2022) and postulate that PEX5 does become inserted into the docking/translocation complex (Gouveia et al. 2003; Miyata and Fujiki 2005; Skowyra and Rapoport 2022). Accordingly, the translocon would be presented as the large and flexible docking/translocation module into which PEX5 enters to release its cargo (Barros-Barbosa et al. 2019). How is this supposed to work?

Besides PEX5, the peroxins PEX14 and PEX13 play an important role in peroxisomal protein translocation (Fig. 1). Upon cargo binding, PEX5 exposes an intrinsically disordered region (IDR), which interacts with PEX13 and PEX14 (Francisco et al. 2017). Furthermore, PEX13 contains a series of conserved Tyr-Gly (YG) repeats within its own IDR. Interestingly, the IDRs resemble those of nucleoporin proteins of nuclear pore complexes (e.g., the nucleoporin FG repeats), suggesting similarities of peroxisomal protein import to nuclear transport, with PEX5 acting as a shuttling receptor analogously to karyopherins (Barros-Barbosa et al. 2019; Gao et al. 2022; Ravindran et al. 2023; Rudowitz and Erdmann 2023). In line with this, purified YG domains were shown to form hydrogels into which PEX5 selectively partitions, using conserved aromatic WxxxF/Y motifs. It is suggested that the YG domains of multiple PEX13 molecules of opposite transmembrane orientations form a meshwork, or selective phase, in the peroxisomal membrane resulting in an aqueous conduit through which PEX5 delivers folded proteins into peroxisomes (Gao et al. 2022) (Fig. 1 top). In this model, PEX13 would form a stable “translocation pore” within the peroxisomal membrane. An alternative model has recently been proposed suggesting that liquid–liquid phase separation of PEX5–cargo with PEX13 and PEX14 results in transient protein transport channels (Ravindran et al. 2023). It is suggested that PEX5–cargo protein complexes phase separate with PEX13 or PEX14 through protein–protein interactions between their IDRs to form condensates that create conduits for cargo release into the peroxisome matrix. *S. cerevisiae* Pex13 liquid condensates were shown to be sufficient for *Sc*Pex5-facilitated partitioning of cargo into the condensate. Fluorescence cross-correlation spectroscopy revealed that *Sc*Pex13 and *Sc*Pex14 can form transient foci at peroxisomal membranes and may form channels at different saturating concentrations of *Sc*Pex5-cargo (Ravindran et al. 2023). The transient nature of the foci formed by *Sc*Pex13 and cargo with a short and heterogeneous lifetime of < 2.2 s may explain why a pore has not yet been visualised. Interestingly, there may also be similarities to protein import into chloroplasts (Ganesan et al. 2018; Ouyang et al. 2020).

An additional role for PEX13 as a novel pexophagy regulator has recently been revealed (Demers et al. 2023). The loss of PEX13 caused an accumulation of ubiquitinated PEX5 on peroxisomes and an increase in peroxisome-dependent ROS that combine to induce pexophagy. These findings indicate that PEX13 is required to prevent the degradation of healthy peroxisomes and that components of the peroxisomal matrix protein import machinery contribute to quality control through the activation of pexophagy.

### Mysterious PEX5 recycling

To start a new import cycle, PEX5 needs to be recycled back to the cytosol. This process requires mono-ubiquitination of PEX5 at a conserved N-terminal cysteine-residue and subsequent extraction by the PEX1-PEX6 ATPase complex (Blok et al. 2015) (Fig. 1). Alternatively, PEX5 can be poly-ubiquitinated on lysine residues and subsequently degraded by the proteasome. This alternative pathway has been named RADAR (receptor accumulation and degradation in the absence of recycling) (Léon et al. 2006). Both mono- and poly-ubiquitination is mediated by PEX2/PEX10/PEX12, three RING finger domain-containing proteins, which form a membrane-embedded ubiquitin ligase (E3) complex (El Magraoui et al. 2012; Okumoto et al. 2014) (Fig. 1; Table 1). Cargo release likely occurs before ubiquitination and extraction of PEX5 (Francisco et al. 2013). New hypotheses about the recycling process have now been proposed based on the cryo-electron microscopy structure of the ligase complex of the thermophilic fungus *Thermothelomyces thermophiles* (Feng et al. 2022). It has been reported that each subunit of the complex contributes five transmembrane segments that co-assemble into an open “retrotranslocation channel” for PEX5 and other peroxisomal import receptors (e.g., PEX7). The three RING finger domains form a cytosolic tower, with the RING finger of 2 (RF2) positioned above the channel pore (Feng et al. 2022). The authors propose that the N-terminus of PEX5 is inserted from the peroxisomal lumen into the potential pore and mono-ubiquitinated by RF2 to enable extraction into the cytosol. In cases where its recycling is compromised, PEX5 is poly-ubiquitinated by the concerted action of RF10 and RF12 and degraded via the RADAR pathway (Feng et al. 2022). These hypotheses may shed new light on how PEX5 re-emerges into the cytosol and how mono- and poly-ubiquitination is mediated. The proposed overall process is similar to the retrotranslocation of misfolded proteins from the ER into the cytosol in ER-associated protein degradation (ERAD) (Wu and Rapoport 2018).

Previous reports have suggested that PEX5 accompanies cargo into the peroxisomal lumen (Dammai and Subramani 2001), but experimental evidence also suggests that PEX5 could stay at the peroxisomal membrane, for example, associated with the docking/translocation machinery. Using a cell-free system based on *Xenopus* egg extract, Skowyra and Rapoport proposed that PEX5 accompanies cargo completely into the peroxisomal matrix, using N-terminal WxxxF/Y motifs that bind a luminal domain of the PEX13/PEX14 docking complex. Export of PEX5 is initiated by binding to the PEX2/PEX10/PEX12 ligase complex through an amphipathic helix (Skowyra and Rapoport 2022). The unstructured N-terminus is then supposed to insert into the ligase “retrotranslocation channel” (see above). It emerges into the cytosol for mono-ubiquitination at the conserved cysteine residue and is then pulled out through the channel by the PEX1/PEX6 AAA ATPase. This is accompanied by the unfolding of PEX5 and cargo release. These findings indicate that PEX5 enters the peroxisome in a folded state but is unfolded during retrotranslocation. Whether PEX5 indeed fully enters the peroxisomal matrix, or whether it is held inside the docking/translocation module in a flexible/dynamic manner (Gouveia et al. 2003; Barros-Barbosa et al. 2019), requires further investigation.

In contrast to the matrix protein import step, which—remarkably—does not require ATP hydrolysis (Alencastre et al. 2009; Francisco et al. 2013), the export of PEX5 depends on ATP hydrolysis mediated by PEX1 and PEX6, two members of the AAA ATPases family, which are anchored to the peroxisomal membrane by PEX26 in mammals (APEM9 in plants; Pex15 in yeast/fungi) (Fig. 1). The structure and function of this receptor-export module has been well characterised in yeast (reviewed in Tan et al. 2016). PEX1 and PEX6 assemble into a ring-shaped hetero-hexameric complex (a trimer of PEX1–PEX6 heterodimers), with a relatively large pore at its centre. Conserved hydrophobic residues of the so-called pore loops are important for PEX1–PEX6 function and substrate handling. It has been reported that the PEX1–PEX6 complex unfolds substrates in a pore loop-dependent manner using a processive threading mechanism (Gardner et al. 2018). Furthermore, ubiquitinated PEX5 interacts directly with PEX1–PEX6 and is globally unfolded during the ATP-dependent extraction step (Pedrosa et al. 2018; Hagmann et al. 2018). The mono-ubiquitin that is attached to PEX5 represents the actual substrate for PEX1/6 (Pedrosa et al. 2023a). The ubiquitin moiety of Ub-PEX5 is recognised and unfolded by PEX1/6; the PEX5 polypeptide chain just hitches a ride during the subsequent processive dislocation process. This establishes a parallel between PEX1/6 and the ERAD-mediating ATPase Cdc48/p97, which also recognises and unfolds its substrates by grabbing and unfolding a ubiquitin molecule that is attached to the substrate.

Very recently, data about the first high-resolution cryo-EM structures of the Pex1/Pex6 complex from *S. cerevisiae* were presented including two different conformations bound to an endogenous substrate. Rüttermann et al. showed that translocation of the substrate occurs along the central pore formed by rings D1 and D2; however, ATP hydrolysis is restricted in the ring D2 (Rüttermann et al. 2023). The pore II loops of the Pex1/Pex6 (D2) subdomains bind the substrate in a canonical staircase arrangement (Puchades et al. 2020), but Pex1 and Pex6 function in pairs. The ATPase cycle involves uncoupling of a “twin seam” Pex1/Pex6 (D2) heterodimer from the staircase. The respective mechanical forces are transmitted to the D1 ring via different interfaces, resulting in alternate widening and constriction of its pore (Rüttermann et al. 2023).

A recent proteomics study has investigated the impact of the reduction of the E1 enzyme UBA1 and several E2 ubiquitin-conjugating enzymes on cellular function (Hunt et al. 2023). Interestingly, profound adaptations in peroxisomes were triggered by decreased ubiquitination in human HEK293T cells. The study revealed that UBA1/E2 knockdown induces the compensatory upregulation of PEX proteins necessary for PEX5 docking to the peroxisomal membrane. The increase in peroxisomal protein import upon global reduction in UBA1/E2 function is supposed to be caused by reduced turnover and subsequent increase in the levels of peroxins required for the docking of PEX5 and peroxisomal matrix protein import.

## Mysterious shapers, movers, and regulators of peroxisome dynamics

**Fig. 3:**
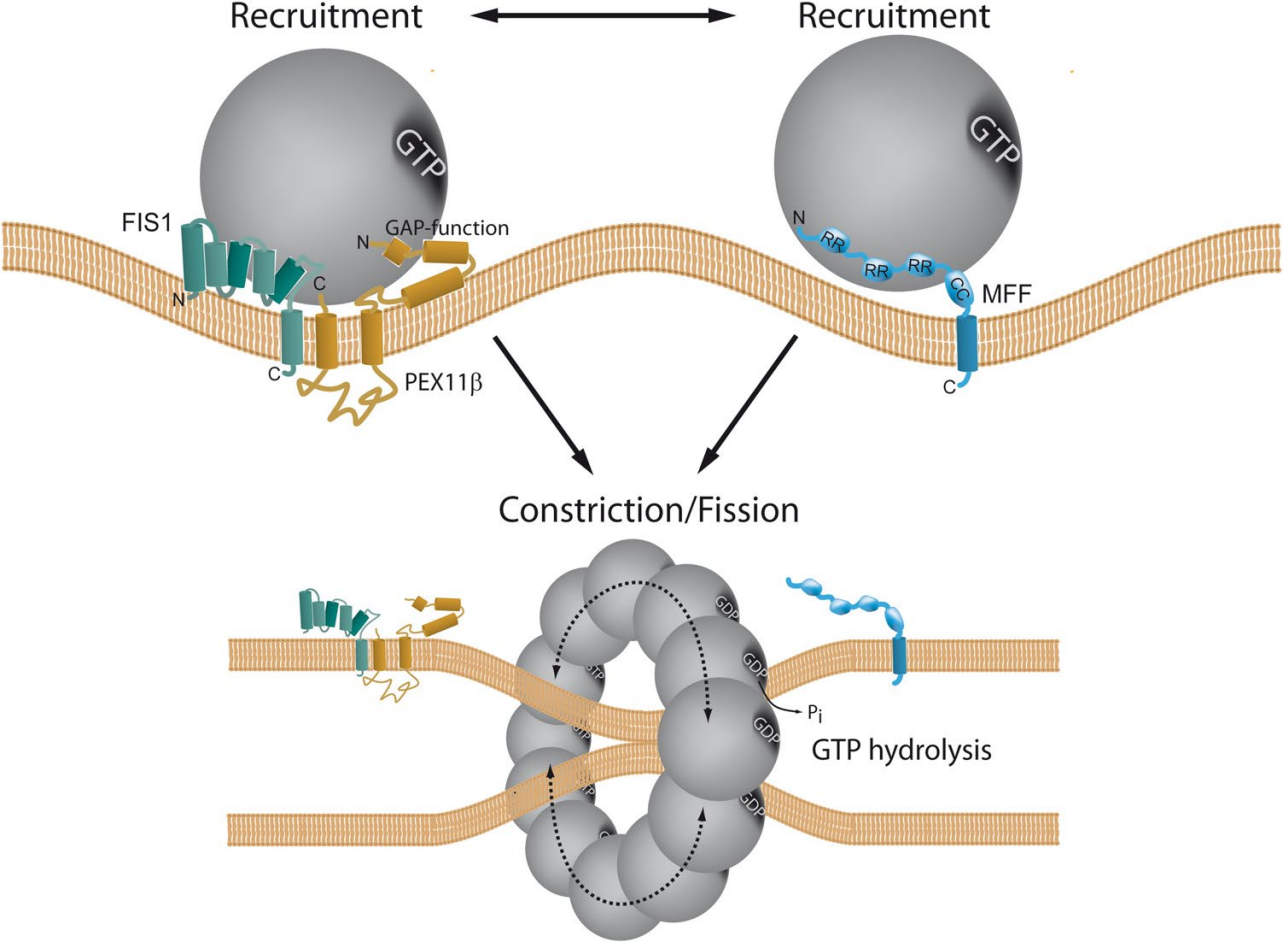
Schematic of the two alternative pathways of peroxisome division, the PEX11β-FIS1-dependent and the MFF-dependent peroxisome division pathway. *CC* coiled-coil region; *GAP* GTPase-activating protein–function; *RR* repetitive region. Taken from Schrader et al. (2022)

Peroxisomes are surprisingly dynamic organelles which can multiply by membrane growth and division of pre-existing organelles (recently reviewed in Imoto et al. 2020; Carmichael and Schrader 2022). This multistep process is initiated by remodelling and expansion of the peroxisomal membrane through the formation of tubular membrane extensions. This is followed by membrane constriction and final membrane scission/division, which gives rise to new peroxisomes. In mammals, membrane expansion is mediated by the peroxisomal membrane-shaping protein PEX11β, which is also involved in the assembly of the fission machinery and activation of the dynamin-like fission GTPase DRP1 (dynamin-related protein 1) (Fig. 1). The fission machinery includes the tail-anchored membrane adaptors MFF (mitochondrial fission factor) and FIS1 (mitochondrial fission protein 1), which can recruit DRP1 to the organelle membrane (Figs. 1 and 3). Once recruited, DRP1 forms oligomeric, ring-like structures at the constriction sites, and upon GTP hydrolysis, final membrane scission is executed through a conformational change of the ring. Remarkably, except for PEX11β, the fission proteins are shared with mitochondria, likely to coordinate peroxisomal and mitochondrial dynamics due to the two organelles’ cooperative functions in fatty acid degradation and ROS homeostasis. Similarities and differences between peroxisomal and mitochondrial division have recently been reviewed (Subramani et al. 2023). Patients with a loss of DRP1, MFF or PEX11β function have been identified who suffer from neurological abnormalities and exhibit elongated peroxisomes (and mitochondria—in DRP1 and MFF deficiency) due to impaired organelle dynamics (recently reviewed in Carmichael et al. 2022).

Additional components which regulate and support the process at peroxisomes such as curvature-generating lipids, lipid-modifying proteins, kinases, or phosphatases likely exist, but await identification. Recently, a supporting role for DYNAMO1 (dynamin-based ring motive-force organiser 1)/NME3 (nucleoside diphosphate kinase 3) in the local supply of GTP for DRP1-mediated fission has been reported (Imoto et al. 2018; Honsho et al. 2020a) (Fig. 1). Interestingly, DYNAMO1 of the red alga *Cyanidioschyzon merolae* and its mammalian ortholog NME3 can be recruited to both mitochondria and peroxisomes. A role in peroxisome division is supported by the observation that peroxisomes are elongated after reduced expression of NME3 and in patient fibroblasts lacking NME3 (Honsho et al. 2020a).

As stated above, loss of the DRP1 membrane adaptor MFF causes the formation of highly elongated peroxisomes. Interestingly, these were observed to consist of long membrane protrusions emanating from a spherical “mother” peroxisome (peroxisome body) in patient fibroblasts (Passmore et al. 2020). Whereas the body represents a mature, import competent structure, the membrane tubules appear to be premature peroxisomes. Thus, the overall number of functional peroxisomes is reduced in patient cells, explaining why peroxisomes are still functional, but may struggle to cope with metabolic challenges. In line with this, a decrease in docosahexaenoic acid (DHA, C22:6)-containing phospholipids was recently reported in fibroblasts from patients deficient in DRP1, NME3, or PEX11β (Abe et al. 2023). In addition to elongated mitochondria and block of mitochondrial fission, DRP1-deficient patient cells also display lower coupling efficiency, increased proton leak, upregulation of glycolysis, aberrant cristae structure and hyperpolarised mitochondrial membrane potential. Mitochondrial respiration is reduced when these cells have to rely on fatty acid oxidation, indicating an involvement of peroxisomes. Furthermore, metabolomic analyses revealed an impairment of the methionine cycle and the synthesis of pyrimidine nucleotides (Robertson et al. 2023).

Whereas MFF is disease-relevant, and its loss causes peroxisome elongation due to a block in fission, patients with a defect in FIS1 have not yet been identified. Furthermore, loss of FIS1 does not result in a prominent peroxisome elongation. These observations have led to the assumption that MFF is the major adaptor protein for peroxisome (and mitochondrial) division, whereas FIS1 may have more specialised functions (Ihenacho et al. 2021). Interestingly, we recently revealed that PEX11β can promote peroxisome division even in MFF-deficient fibroblasts (Schrader et al. 2022). The PEX11β-mediated peroxisome division in the absence of MFF depends on DRP1 and FIS1. Conversely, MFF can promote peroxisome division in PEX11β-deficient fibroblasts and restore the normal, spherical peroxisome morphology. The division-promoting activity of PEX11β was dependent on amino acids at its C-terminus, which may be essential for the formation of an active division complex with FIS1 and DRP1. A conserved, non-canonical insert in FIS1 appears to be required for DRP1 recruitment and organelle fission (Ihenacho et al. 2023). Interestingly, alterations of the N-terminus did not prominently impact on the ability of PEX11β to induce peroxisome division. However, the N-terminus, with its amphipathic helices, is crucial for peroxisome membrane elongation. Overall, these findings demonstrate a function for FIS1 in peroxisome division and indicate that two alternative pathways for peroxisome division exist: an MFF-dependent and an alternative PEX11β/FIS1-dependent pathway (Fig. 3). As MFF is only found in metazoa, we speculate that the PEX11β/FIS1-dependent pathway may be the evolutionary ancient one. An additional MFF-dependent mechanism may have evolved in animals due to the increasing functional cooperation of peroxisomes and mitochondria in fatty acid β-oxidation, ROS homeostasis and antiviral defence. As MFF is targeted to both peroxisomes and mitochondria, it may allow the coordinated division of both organelles, whereas PEX11β, which is only peroxisomal, would allow regulation of peroxisome division independent of mitochondria according to metabolic needs (Schrader et al. 2022). Future studies will be necessary to reveal what the physiological functions of those alternative division pathways are.

PEX11β can deform and elongate the peroxisomal membrane through amphipathic helices in its N-terminus, which can interact with membrane lipids, and through homo-oligomerisation (reviewed in Carmichael and Schrader 2022). However, membrane elongation also depends on the supply of phospholipids. It has been shown that phospholipid transfer from the ER to peroxisomes depends on peroxisome-ER MCSs involving the peroxisomal membrane proteins ACBD5 and ACBD4, which can interact with ER-resident VAP proteins (Costello et al. 2017b; Hua et al. 2017) (Fig. 1). ACBD4/5-VAP tethering is mediated by a FFAT (two phenylalanines in an acidic tract) motif that interacts with an MSP domain of VAP. Loss of ACBD5 or VAP reduces peroxisomal membrane expansion indicating that close proximity of the ER and peroxisome membranes is important for lipid transfer. Although ACBD5 possesses a fatty acid binding domain at its N-terminus, this domain is likely not involved in phospholipid transfer, as an artificial peroxisome-ER tether can promote peroxisomal membrane expansion in the absence of ACBD5 (Costello et al. 2017b). This need for peroxisome-ER tethering for peroxisomal membrane expansion and thus biogenesis is conserved within eukaryotes, with membrane surface area and peroxisome number being reduced in *H. polymorpha* cells lacking the ER-resident tether proteins Pex24 and Pex32 (Table 1). Importantly, however, similar to the case of ACBD5, these phenotypes can be rescued by artificial peroxisome-ER tethering(Yuan et al. 2022).

Recently, a role for VPS13D in peroxisome biogenesis has been reported (Baldwin et al. 2021). VPS13D belongs to a family of large channel-forming lipid transfer proteins, which are supposed to bridge membranes and allow “bulk flow” of lipids through a long hydrophobic groove from the ER to other organelles (Neuman et al. 2022) (Fig. 1). Interestingly, VPS13D can interact with ER-resident VAP proteins, likely through a FFAT motif. Furthermore, VPS13D can be recruited to peroxisomes and mitochondria via MIRO1, a tail-anchored membrane adaptor protein for microtubule-dependent motor proteins (Guillén-Samander et al. 2021). MIRO1 appears to be dually localised to peroxisomes and mitochondria (Castro et al. 2018; Okumoto et al. 2018; Covill-Cooke et al. 2020; Covill-Cooke et al. 2021; Zinsmaier 2021) (Fig. 1). It is therefore likely that VPS13D can bridge the ER and peroxisomes via interaction with VAP and MIRO1 to allow “bulk flow” of lipids for membrane expansion. In support of this notion, loss of VPS13D or Vps13 in yeast has been reported to impact on peroxisomal membrane expansion (Yuan et al. 2022; Pedrosa et al. 2023b). The MIRO1/motor complex has also been shown to exert pulling forces at peroxisomes, which can result in dynamic peroxisome tubule formation and membrane expansion (Castro et al. 2018). The N-terminal GTPase domain of MIRO1 appears to be critical for the regulation of its interaction with the components of the motor–adaptor complex and regulation of organelle motility (Davis et al. 2022). As the surface area of those tubules is much larger than the peroxisome body they emanate from, their formation would require phospholipid supply from the ER. The highly dynamic nature of these processes would also be in support of a “bulk flow” mechanism for lipids. We speculate that MIRO1 may coordinate lipid flow from the ER to peroxisomes (via interaction with VPS13D) with peroxisomal tubule formation/membrane expansion (via its interaction with motor proteins and force generation along microtubules). Additionally, it is likely that other components such as PEX11β are involved in the regulation of these processes, as loss of PEX11β impairs MIRO1/motor complex-mediated tubule formation (Kustatscher et al. 2019).

Furthermore, a role for the kinase GSK3β in the phosphorylation of ACBD5 and regulation of peroxisome-ER tethering, and thus peroxisome membrane elongation, has been revealed (Kors et al. 2022) (Fig. 1). GSK3β directly phosphorylates a serine residue in the core region of the ACBD5 FFAT motif, which results in an inhibition of the interaction with ER-resident VAPB and reduced peroxisome-ER tethering. However, phosphorylation of serine/threonine residues in the acidic tract adjacent to the core of the FFAT motif is required for ACBD5-VAPB interaction. The complex regulatory mechanism supports a two-step model of ACBD5-VAPB interaction: (i) phosphorylation of the acidic tract is required to support interaction of the FFAT motif with the basic electropositive face of the VAPB MSP domain; (ii) the FFAT core region then needs to bind in a hydrophobic pocket of the MSP domain. This second step is inhibited when the FFAT core region is phosphorylated. These findings also apply to the interaction of other FFAT-containing proteins with MSP domains (Kors et al. 2022).

The regulation of ACBD5-VAP mediated peroxisome-ER tethering is one of the few examples demonstrating a physiological role of phosphorylation in mammalian peroxisome biogenesis. Another recent example is the phosphorylation of PEX14, a crucial component of the peroxisomal import machinery (Fig. 1). Phosphorylation of mammalian PEX14 in response to oxidative stresses such as H_2_O_2_ suppresses the peroxisomal import of catalase, which is involved in the degradation of H_2_O_2_. This mechanism is supposed to counteract cellular oxidative stress by increasing catalase in the cytosol (Walton et al. 2017; Okumoto et al. 2020) and to protect DNA upon nuclear envelope breakdown during mitosis (Yamashita et al. 2020). In earlier studies, phosphorylation of human PEX5, the shuttling import receptor for peroxisomal matrix proteins (Fig. 1) has been linked to pexophagy in response to ROS (Zhang et al. 2015). Additionally, Pex14 phosphorylation in the yeast *S. cerevisiae* controls the import of Cit2, the peroxisomal isoform of citrate synthase (Schummer et al. 2020). Earlier studies in yeast also provide insight into the physiological role of phosphorylation of peroxisomal proteins. In *S. cerevisiae* and *Pichia pastoris*, the peroxisome biogenesis factor Pex11 is activated by site-specific phosphorylation (Knoblach and Rachubinski 2010; Joshi et al. 2012), although this appears to be different in *H. polymorpha* (Thomas et al. 2015). In addition, pexophagy requires the phosphorylation of pexophagy receptor Atg30 in *P. pastoris* (Burnett et al. 2015).

## Progress towards understanding the mysteries of peroxisome-organelle membrane contact sites

Peroxisomes are now well established to be “social” organelles requiring close physical contact with other subcellular organelles (including the ER, LDs, mitochondria, and lysosomes) at membrane contact sites (MCSs) to fulfil their various metabolic and non-metabolic functions (Scorrano et al. 2019; Prinz et al. 2020; Wanders et al. 2023). Due to the increasing appreciation of the role that inter-organelle communication plays in cellular physiology, peroxisome MCSs have been extensively reviewed in recent years, in terms of composition, regulation, and physiological function, in both yeast (Wu et al. 2023) and mammals (Chen et al. 2020; Silva et al. 2020). Here, we provide a brief update on the most recent discoveries regarding the mechanisms and function of peroxisome-organelle interactions at MCSs, as well as the development of novel techniques to quantify contacts and identify novel tethering components.

### New methods to address the mysteries of peroxisome-organelle contacts

New technical developments in both the quantification of MCSs and the unbiased identification of new proteins residing at and/or regulating MCSs, have been instrumental in driving recent advances in our understanding of peroxisome-organelle contact sites. While EM remains the “gold standard” for assessing physical organelle interactions, as it has the resolution to detect membranes in close-enough apposition to be defined as an MCS, the optimisation of fluorescence-based techniques including proximity ligation assays and bimolecular fluorescence complementation for studying peroxisomal interactions has made the quantification of MCSs and screening for conditions and components that regulate them more accessible (Bishop et al. 2019; Huang et al. 2020; Vallese et al. 2020). Recently, high-throughput microscopy screening of *S. cerevisiae* using split fluorescent protein-tagged organelle reporters (Shai et al. 2018) has been combined with yeast libraries overexpressing fluorescently tagged proteins to systematically identify novel MCS tethers and regulators through colocalisation imaging and quantification of reporter signal number/intensity (Castro et al. 2022). This screen identified over 100 new potential MCS resident and effector proteins in yeast, including the previously uncharacterised and highly dynamic Golgi-peroxisome MCS. Interestingly, while Golgi-peroxisome MCSs are infrequently observed under normal conditions, their number increases upon amino acid starvation, implying they are metabolically regulated (Castro et al. 2022). Overall, this study has demonstrated that there are many MCS proteins yet to be discovered or characterised and provides an invaluable resource identifying novel targets for future physiological investigation.

### More mysteries solved concerning the components and functions of peroxisome-organelle cooperation

Following on from pioneering studies observing the diversity of peroxisome-organelle MCSs (Valm et al. 2017; Shai et al. 2018), subsequent work has focused on characterising the composition, regulation, and physiological roles of these contacts. Below, we summarise some of the most recent advances concerning the properties and functions of specific peroxisome-organelle MCSs.

#### Peroxisome-ER MCSs

**Fig. 4:**
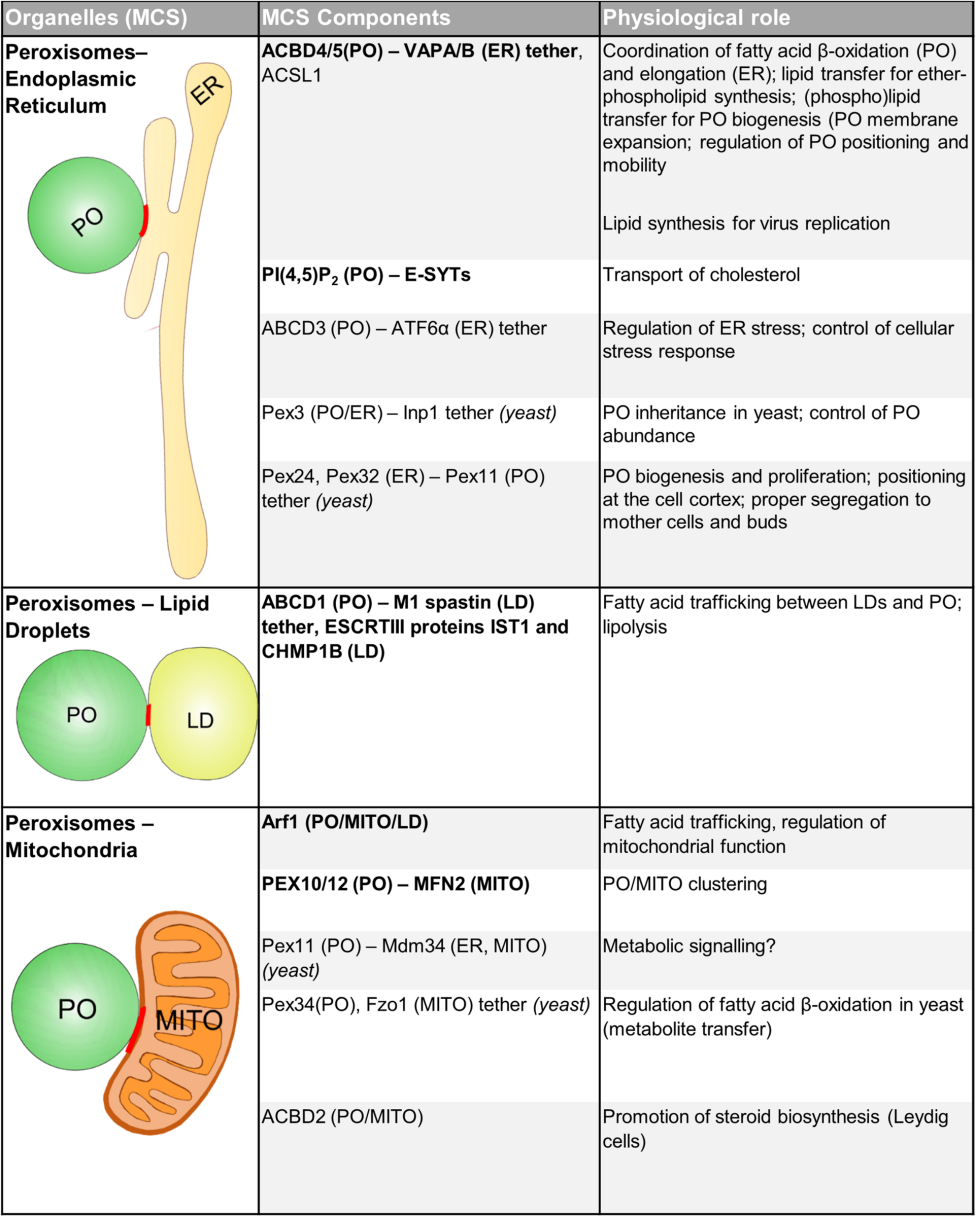
Known components of peroxisome-organelle MCSs. For details see text and Silva et al. (2020). *ABCD1/3* ATP-binding cassette sub-family D member 1/3; *ACBD2/4/5* acyl-coenzyme binding domain protein 2/4/5; *ACSL1* long chain fatty-acid-CoA ligase 1; *Arf1* ADP-ribosylation factor 1; *ATF6α* activating transcription factor 6α; *CHMP1B* charged multivesicular body protein 1B; *ER* endoplasmic reticulum; *ESCRTIII* endosomal sorting complexes required for transport III; *Fzo1* fuzzy onions homolog 1; *Inp1* inheritance of peroxisomes protein 1; *IST1* vacuolar protein sorting-associated protein 1; *LD* lipid droplet; *Mdm34* mitochondrial distribution and morphology protein 34; *MFN2* mitofusin 2; *MITO* mitochondrion; *M1* spastin, isoform M1 of the microtubule-severing protein spastin; *Pex* peroxin; *PI(4,5)P2* phosphatidylinositol-4,5-biphosphate; *PO* peroxisome; *E-Syts* ER-resident extended synaptotagmins; *VAPA/B* vesicle-associated membrane protein (VAMP)-associated protein A/B. MCS proteins are mammalian unless otherwise stated, and those in bold are addressed further in the text. Adapted from Silva et al. (2020)

Peroxisome-ER tethering, mediated via the ACBD5-VAPB tether protein interaction in mammals (Figs. 1 and 4) (see “Mysterious shapers, movers, and regulators of peroxisome dynamics”), is important for human health, given the pathologies observed in ACBD5-deficient patients (Carmichael et al. 2022). An ACBD5-deficient mouse model (*Acbd5*^*−/−*^) has recently been developed to characterise the pathological changes in various tissues, providing a better understanding of how loss of ACBD5 leads to disease symptoms (Darwisch et al. 2020). The *Acbd5*^*−/−*^ mouse recapitulates aspects of the human phenotype, displaying progressive cerebellar and retinal degeneration. Lipid profiles are dysregulated in a tissue-specific manner compared to controls, including increased ultra-long-chain fatty acid levels, but reduced ether phospholipid levels, in the cerebellum. Interestingly, some of these lipid alterations seen in *Acbd5*^*−/−*^ mice differ from those seen in β-oxidation or very-long-chain fatty acid (VLCFA) import single-enzyme deficiencies, being instead more similar to ZSD patient profiles (Darwisch et al. 2020). Together, this suggests these changes are attributable to defects in peroxisome-ER tethering rather than just ACBD5’s role in channelling VLCFAs into the peroxisome, supporting the hypothesis that ACBD5 acts as part of a metabolic hub linking peroxisomes and the ER.

One of the key roles of peroxisome-ER MCSs is to facilitate ER to peroxisome phospholipid flow to support the peroxisomal membrane expansion required for biogenesis (see “Mysterious shapers, movers, and regulators of peroxisome dynamics”). A second form of lipid transfer between peroxisomes and the ER has since been identified, but in the opposite direction, with cholesterol being able to be shuttled from the lysosome, where it is first trafficked after internalisation, via peroxisomes, to the ER, where it is required for structure and function (Xiao et al. 2019). The transfer of cholesterol from peroxisomes to the ER occurs at MCSs formed through tethering of the ER-resident extended synaptotagmins (E-Syts) to PI(4,5)P_2_ in the peroxisomal membrane, with depletion of either component resulting in reduced peroxisome-ER contacts, and knockdown of E-Syts increasing cholesterol accumulation in the lysosome. In vitro assays measuring transfer of radiolabelled cholesterol between purified peroxisomes (or artificial liposomes containing defined PI(4,5)P_2_ levels) and ER/microsomes confirmed a decrease in peroxisome-to-ER cholesterol trafficking in the absence of E-Syts or peroxisomal/liposomal PI(4,5)P_2_. Furthermore, supporting a role for peroxisomes as a conduit for cholesterol trafficking from lysosomes to the ER, triple ER-peroxisome-lysosome contacts are observed in HeLa cells, with the addition of purified peroxisomes to purified lysosomes and ER/microsomes enhancing lysosome to ER cholesterol transport in vitro in a PI(4,5)P_2_-dependent manner (Xiao et al. 2019)**.**

#### Peroxisome-lipid droplet MCSs

As both are key lipid-metabolising organelles, peroxisomes must cooperate tightly with LDs, which are highly dynamic and consist of a core of neutral lipids surrounded by a single phospholipid layer (Walther and Farese 2012; Welte 2015; Olzmann and Carvalho 2019). The close relationship between these organelles is demonstrated by their co-regulation, for example both peroxisomes and LDs increase in number in response to monounsaturated fatty acid exposure in *Caenorhabditis elegans*, which is important for longevity (Papsdorf et al. 2023). Fatty acids must be trafficked between peroxisomes and LDs depending on cellular demands—either routed to peroxisomes to be β-oxidised for energy generation or routed to LDs for storage when in excess. MCSs are reported to be formed between peroxisomes and LDs in mammalian cells through the interaction between the LD membrane-bound protein M1 spastin and the peroxisomal fatty acid transporter ABCD1 (Figs. 1 and 4), and this tether has been proposed to mediate fatty acid trafficking between these two organelles. M1 spastin recruits the membrane-shaping ESCRT-III proteins IST1 and CHMP1B to LDs, which may play a role in modifying the phospholipid monolayer to support efficient fatty acid trafficking between peroxisomes and LDs (Chang et al. 2019). Physical contact between peroxisomes and LDs has also been shown to promote lipolysis (the breakdown of triacylglycerols stored in LDs into free fatty acids that can be utilised for energy generation) in *C. elegans* and mice. Upon fasting, colocalisation of peroxisomal markers with LDs increases, leading to recruitment of the triacylglycerol hydrolase ATGL from the cytosol to these points of contact via PEX5 (Kong et al. 2020). Both PEX5 and peroxisome-LD contact are essential for fasting-induced lipolysis, as this process is occluded by either PEX5 knockdown or microtubule depolymerisation, which prevents peroxisomes being transported to LDs. A link between peroxisomes and LDs has also been observed in *Drosophila* (Ueda et al. 2022). The peroxins Pex3, Pex13, and Pex14 were observed to surround newly formed LDs. The reduction of these LD-associated peroxins caused a unique effect on larval fat body development, indicating a non-canonical role of these peroxins in the regulation of lipid storage.

Furthermore, it has been proposed that the peroxisome bilayer and the LD monolayer may undergo hemifusion at points of physical contact to promote fatty acid transfer, with peroxisomes in yeast and plants being observed to form extensions called “pexopodia” that reach into the LD core and favour β-oxidation (Binns et al. 2006; Thazar-Poulot et al. 2015). Recent molecular dynamics modelling approaches have revealed the possible dynamic basis of this hemifusion, with the formation of the hourglass-shaped “stalk” that arises from the initial monolayer fusion being energetically driven by the incorporation of free fatty acids possessing negative spontaneous curvature into the stalk, which allows its radial expansion into a “pexopodium” (Kalutsky et al. 2022).

#### Peroxisome-mitochondria MCSs

It is widely accepted that peroxisomes and mitochondria have a close functional relationship, with both sharing components of their division machinery, and having cooperative roles in β-oxidation of fatty acids and ROS homeostasis (Schrader and Yoon 2007; Fransen et al. 2017; Costello et al. 2018). However, despite this old adage, peroxisome-mitochondria contacts are observed much less frequently than peroxisome-ER contacts (Valm et al. 2017), although this may reflect a more dynamic nature. Additionally, new mechanisms of peroxisome-mitochondria metabolic interplay are still being uncovered. For example, generation of PI(4,5)P_2_ at the peroxisomal membrane by phosphatidylinositol-5-phosphate 4-kinases (PI5P4Ks) drives trafficking of VLCFAs into peroxisomes, which in turn is important for mitochondrial function, with loss of PI5P4Ks significantly disrupting mitochondrial lipid uptake, β-oxidation, and structural/functional integrity (Ravi et al. 2021). Metabolic interplay between peroxisomes and mitochondria can also require wider cooperation with other organelles. Three-way contacts have been observed at an ultrastructural level between the ER, peroxisomes, and mitochondria in mouse liver, with peroxisomes recruited to these contacts in response to fasting or increased β-oxidation, suggesting a role in maintaining liver lipid homeostasis (Ilacqua et al. 2021). Furthermore, the small GTPase Arf1 localises to contacts between peroxisomes, mitochondria, and LDs in yeast, regulating the flow of fatty acids from LDs to mitochondria/peroxisomes and ultimately controlling mitochondrial function (Enkler et al. 2023) (see “Mysterious functions: peroxisomal metabolism updated”). Finally, whilst peroxisome-mitochondria MCSs have been characterised in yeast (Mattiazzi Ušaj et al. 2015; Shai et al. 2018; Joshi 2021) (Fig. 4) their composition and regulation remains more mysterious in mammals. A potential new mediator of mammalian peroxisome-mitochondria contacts was revealed using a proximity labelling assay to reveal proteins adjacent to the peroxisomal ubiquitin ligase complex of PEX2/10/12 in HeLa cells. This identified the mitochondrial fusion proteins MFN1/2 in close proximity to peroxisomes, with MFN2 being observed to be enriched at points of peroxisome-mitochondria contact (Huo et al. 2022).

Overall, recent advances have begun to reveal some of the previously unknown functions, composition, and regulation of peroxisome-organelle MCSs. What remains a mystery, and is therefore the subject of ongoing investigation, is the contribution of these MCS functions in maintaining cell health under physiological conditions, and to what extent peroxisome-MCS dysregulation contributes to pathologies.

## Mysterious new roles for peroxisomes in immune and defence mechanisms

As recently as a decade ago, peroxisomes were not known to be associated with immunometabolism, but are now emerging as one of the key organelles contributing to host–pathogen interactions, antiviral responses, and regulation of immune signalling (Di Cara 2020; Ferreira et al. 2022). Interestingly, the metabolic activities of peroxisomes, such as ɑ- and β-oxidation of fatty acids, plasmalogen synthesis, and ROS/reactive nitrogen species (RNS) metabolism, have been linked to numerous immune-related pathways (reviewed in Di Cara et al. 2019; Islinger et al. 2018). Recent studies have explored peroxisome biology during host–pathogen interactions, from roles in fighting infection to being hijacked to serve viral replication (Ferreira et al. 2022). A recent review from Di Cara et al. has extensively discussed the profound relationship between peroxisomes and immunometabolism (Di Cara et al. 2022).

### Peroxisomal functions regulate immune responses

Peroxisomal β-oxidation of VLCFAs plays a significant role in maintaining the intracellular homeostasis of fatty acids, which are crucial regulators of immune responses, acting as precursors for various phospholipids and polyunsaturated fatty acids (PUFAs). Phospholipids and PUFAs are required to ensure the activation and function of key immune cells such as macrophages, invariant natural killer T cells, and T-helper cells during inflammatory processes (Yaqoob 2003). Peroxisomal β-oxidation also contributes to the synthesis of DHA and helps control the endogenous levels of eicosapentaenoic acid (EPA) and docosapentaenoic acid (DPA), which are crucial for the production of anti-inflammatory and immunoregulatory lipids such as resolvins, maresins, lipoxins, elovanoids, and protectins (Di Cara et al. 2019; Wanders et al. 2023). Several recent studies have implicated peroxisomal β-oxidation in the process of immune cell activation. In *Drosophila* and murine macrophages lacking *Pex2*, and thus functional peroxisomes, the total amount and cellular distribution of glycerophospholipids (whose levels are regulated by peroxisomal β-oxidation) was altered during immune activation in response to pro-inflammatory stimuli, which in turn reduced cytokine secretion (Nath et al. 2022). Similarly, mast cells (immune effectors) from *Pex2*-knockout mice also displayed lower cytokine release when challenged with immunogenic stimuli compared to controls, as a result of free fatty acids accumulating rather than being degraded in peroxisomes (Meghnem et al. 2022). Overall, this suggests that peroxisomal β-oxidation is necessary for the normal inflammatory signalling cascades required for immune cell activation in response to extracellular stimuli.

In addition, peroxisomes cooperate with the ER for ether phospholipid synthesis (Wanders et al. 2023). Ether phospholipids, which are enriched in immune cells such as macrophages and neutrophils, have been linked to inflammation, being rich in PUFAs, DHA and arachidonic acid, which can be released upon plasmalogen hydrolysis. Arachidonic acid is a precursor for bioactive lipids, which are crucial for modulating inflammation, including EPA-dependent pro-inflammatory factors such as prostaglandins, leukotrienes, and thromboxanes, and is also important for the induction of anti-inflammatory and immunosuppressive effects in certain cell types (Savary et al. 2012; Di Cara et al. 2022). Immune cell behaviour is also controlled by prostaglandins and thromboxanes, which are involved in the activation of CD4+ and CD8+ T effector cells and the formation of memory T cells (Maseda et al. 2019). Together, peroxisomal β-oxidation and ether phospholipid synthesis play a major role in the inflammatory response via production of anti- or pro-inflammatory lipid mediators, depending on the cellular context (Savary et al. 2012; Rubio et al. 2018; Bozelli et al. 2021).

ROS/RNS are emerging as important factors for cellular and systemic immune responses, through the activation of immune cells, suppression of the immune response, and rearrangement of the cytoskeleton for phagocytosis (Fransen et al. 2012; Yang et al. 2013). Through their function in maintaining ROS/RNS homeostasis, peroxisomes play a major role in microbe engulfment and host defence by macrophages, by regulating organisation of the phagosome, progression of phagocytosis, and activation of immune responses including the production of antimicrobial peptides (reviewed in Islinger et al. 2018; Terlecky et al. 2012). A reduction in peroxisome function impairs the overall formation of the “oxidative burst” which is crucial for fighting infection. *Drosophila* macrophages lacking functional peroxisomes, as a result of *Pex5* or *Pex7* RNAi treatment, displayed a higher basal hydrogen peroxide (H_2_O_2_) level, but a lower level of H_2_O_2_ induction in response to bacterial infection, compared with control cells with normal peroxisomal function, demonstrating functional peroxisomes are required for H_2_O_2_ turnover which in turn promotes pathogen engulfment (Di Cara et al. 2017).

### Peroxisomes mysteriously help and hinder pathogen infection

Peroxisomes play a role in antiviral signalling and defence via the mitochondrial antiviral signalling protein (MAVS, also known as IPS-1, Cardif, or VISA), a tail-anchored protein which localises to both mitochondria and peroxisomes (Fig. 1), inducing an antiviral cellular response from both platforms (reviewed in Ferreira et al. 2022). Antiviral signalling pathways depend on the detection of viral proteins, genetic material, lipids, lipoproteins, and/or glycan molecules by pattern recognition receptors (PRRs). When viral genetic material is recognised by PRRs, it triggers downstream signalling via MAVS and/or stimulation of interferon (IFN) expression (Ferreira et al. 2019). Peroxisome-associated MAVS signalling induces a rapid but short-lived type-I IFN-independent expression of IFN-stimulated genes, whereas mitochondrial MAVS triggers a slow but sustained induction of IFN-stimulated genes which is type I IFN-dependent (Dixit et al. 2010). Peroxisomes also promote expression of the type-III IFN upon peroxisomal MAVS activation (Bender et al. 2015), which is crucial for antiviral immunity and important for the elimination of microbes (Di Cara et al. 2019; Chathuranga et al. 2021; Ferreira et al. 2022).

In addition, besides playing a major role in antiviral defence, peroxisomes can also be hijacked by viruses to escape the immune response or promote viral replication using different mechanisms depending on the type of virus (Wong et al. 2018, 2019; Xu et al. 2020; Ferreira et al. 2022). For instance, flaviviruses (such as Zika virus [ZIKV], West Nile, and dengue) are found to downregulate peroxisomal biogenesis in their host, with the viral capsid protein of Flaviviruses interacting with PEX19 to drive its sequestration and/or degradation. The reduction in available PEX19 results in a subsequent loss of peroxisomes in virus-infected cells, which therefore reduces the peroxisomal antiviral response, including lower IFN induction (You et al. 2015). Accordingly, promoting peroxisome biogenesis in ZIVK-infected cells through over-expression of PEX11β, to rescue peroxisome numbers, inhibits ZIVK replication and enhances the innate immune response (Wong et al. 2019).

In contrast, some viruses, including human cytomegalovirus (HCMV) and herpes simplex virus type 1 (HSV-1) instead upregulate peroxisome biogenesis through growth and division, which increases the cell’s capacity for ether phospholipid synthesis (Jean Beltran et al. 2018). Surprisingly, despite reduced peroxisome biogenesis, increased levels of ether phospholipids conjugated to PUFAs are also detected in the serum lipidome of ZIKV-infected patients (Queiroz et al. 2019). Since plasmalogens are key components of the viral envelope, boosting peroxisomal metabolism to increase ether phospholipid synthesis is thought to promote the infection cycle and viral particle formation in these cases. Hepatitis C (another member of the Flaviviruses) also exploits peroxisomal lipid metabolism to promote viral propagation, but by inhibiting rather than enhancing it. Liver cells infected with hepatitis C virus showed a downregulation of genes involved in peroxisome function, and a consequent accumulation of VLCFAs due to reduced peroxisomal β-oxidation, with the virus potentially taking advantage of these VLCFAs to promote viral particle assembly (Lupberger et al. 2019). In a recent study, peroxisome function and morphology was reported to be altered in hepatitis C virus-infected cells (Martin de Fourchambault et al. 2023). This included an increase in ROS in peroxisomes and a decrease in catalase activity. However, peroxisomal β-oxidation was not observed to be significantly affected by hepatitis C virus infection in this study. Furthermore, the absence of peroxisomes had no impact on replication kinetics or infectious hepatitis C virus titres (strains JFH1 and DBN3a) in Huh-7 cells. The authors suggest that the peroxisomal perturbations may contribute to liver dysfunction and pathogenesis in chronically infected patients.

Recently, SARS-CoV-2 was reported to induce profound remodelling of several organelles, including peroxisomes, in infected host cells (Cortese et al. 2020; Knoblach et al. 2021). It was observed that peroxisomes accumulate in close proximity to the double membrane vesicles, which are generated upon viral infection and are thought to contribute to replication. The spatial proximity between peroxisomes and these sites of viral RNA replication suggests peroxisomes may play a role in SARS-CoV-2 replication, most likely to protect the viral RNA from potential oxidative damage or to exploit peroxisomal lipid metabolism to facilitate successful replication (Cortese et al. 2020; Knoblach et al. 2021; Hasankhani et al. 2023). In addition, it was found that SARS-CoV-2-infected cells showed a rearrangement of peroxisomal membranes, which become elongated and aggregated, as well as mislocalisation of peroxisomal matrix proteins to the cytosol, resulting in a reduced number of fully functional peroxisomes, and correspondingly an increase in empty “ghost” peroxisomal membranes (Knoblach et al. 2021). This import defect may stem from the reported interaction between the SARS-CoV-2 ORF14 protein and PEX14, which is crucial for peroxisome matrix protein import (Fig. 1). This suggests that SARS-CoV-2 infection leads to peroxisome damage, and thus reduces their ability to perform antiviral signalling (Knoblach et al. 2021).

The *Mycobacterium tuberculosis* (*Mtb*) bacterium is an obligate intracellular pathogen that cannot be destroyed by phagocytosis into macrophages because it modulates phagolysosome biogenesis. Peroxisome proliferator-activated receptors (PPARs PPAR-alpha, PPAR-beta/delta, and PPAR-gamma) are known to play a role in *Mtb* infection. PPAR-alpha is vital for the immune response against *Mtb* infection, as depletion of PPAR-alpha in macrophages resulted in increased growth of *Mtb* by interfering with NF-kB signalling and pro-inflammatory cytokine production, as well as suppressing regulators of autophagic pathways (Ganguli et al. 2019). During *Mtb* infection, there is an increase in the production of oxidative molecules, leading to oxidative stress in the infected cell, which combats the infection by causing damage to the bacterial cell membrane, DNA, and proteins (Nambi et al. 2015). However, *Mtb* acetyltransferase induces the expression of peroxins such as PEX11, PEX5, PEX19, PEX14 and PMP70 (Fig. 1) in infected mouse macrophages, thereby increasing peroxisome biogenesis to successfully neutralise the oxidative stress (Behera et al. 2022). In contrast, Pellegrino et al. (2023) revealed that the increase of peroxisomal number triggered by *Mtb* requires the ESX-1 type 7 secretion system, critical for cytosolic access of *Mtb*. Furthermore, the peroxisomes themselves were observed to underlie the increased ROS levels during *Mtb* infection, suggesting a role for peroxisomes as a source of ROS, particularly H_2_O_2_ production, in the cytosol to restrict *Mtb* replication. The authors therefore suggest a ROS-dependent anti-mycobacterial function for peroxisomes in the cytosol of human macrophages (Pellegrino et al. 2023). This highlights the importance of peroxisomes in controlling *Mtb* infection and suggests they may be potential therapeutic targets for tuberculosis and other bacterial infections (Shastri et al. 2018; Ganguli et al. 2019).

In summary, since peroxisomes are dynamic and complex organelles fulfilling various functions, it is not surprising that peroxisomes regulate the cellular response to infection both positively and negatively, for example, by producing important bioactive compounds to drive immune signalling; by being hijacked to provide metabolites favouring viral replication; and by recruiting antiviral signalling proteins to target membranes. Many outstanding questions are yet to be answered regarding the role of peroxisomes in immunity, particularly their contribution to adaptive immunity and immune cell differentiation, and whether their roles in immune signalling are direct (e.g., as signalling platforms) or indirect (e.g., by altering metabolism and subsequently membrane lipid composition) (Di Cara et al. 2022). Based on their central role, peroxisomes could be new therapeutic targets to treat infections and/or immune disorders, but this remains to be tested.

## Unravelling the mysteries of peroxisomes in discrete places

While peroxisomes are ubiquitous organelles found in almost all tissues, the advent of precision transcriptomics/proteomics and cell type-specific genetic modification, including in animal models, has begun to reveal how peroxisomes are specialised within different tissues, and how they contribute to maintaining organ-specific functions (Wanders et al. 2023). As the organs most obviously affected by peroxisomal disorders, the roles of peroxisomes in the liver and brain are most studied and have been comprehensively reviewed previously (Baes and Van Veldhoven 2015; Berger et al. 2016; Wanders et al. 2023). Here, we consider the most recent developments in our understanding of peroxisome function in different cell/tissue types, in particular their contribution to organ-specific pathologies.

### Heart

Peroxisomes localise close to the T-tubular system and sarcoplasmic reticulum in primary cardiac myocytes (Hicks and Fahimi 1977), or close to the ER in human induced pluripotent stem cell-derived cardiomyocytes (Sargsyan et al. 2021). This localisation fits with the recently proposed role for peroxisomes in the cellular handling of calcium (see “Mysterious functions: peroxisomal metabolism updated”), which is especially important in the heart for coupling electrical stimulation to contraction (Sargsyan et al. 2022). Peroxisomes in both rodent and human cardiomyocytes have been shown to take up and store Ca^2+^ that has been released from intracellular stores in response to cell depolarisation, with both cytosolic and peroxisomal Ca^2+^ levels oscillating in a beat-by-beat manner in response to electrical stimulation (Sargsyan et al. 2021). This implies that peroxisomes could play a role in supporting normal excitation–contraction coupling in the heart. Supporting this, cardiac arrhythmia has been observed in some mild ZSD and Refsum disease patients (Waterham et al. 2021; Bose et al. 2022). However, this symptom has also been recently reported in human patients with a deficiency in the peroxisomal β-oxidation enzyme SCPx (Galano et al. 2022), suggesting peroxisomal fatty acid metabolism may also be important for normal heart contractions.

Manipulation of peroxisome number and/or function has also implicated this organelle in the regulation of other aspects of cardiomyocyte function. Metabolomic/proteomic analysis of heart tissue from *Pex11α*-knockout mice shows dysregulation of processes including glycerophospholipid metabolism, fatty acid processing, steroid biosynthesis, electron transport chain function and ROS metabolism, compared to wild-type animals (Garikapati et al. 2022). Supporting a protective role of peroxisomes in the heart, peroxisome biogenesis is observed to be suppressed in both in vitro and in vivo mouse models of cardiac hypertrophy. Overexpression of PEX5 in in vitro cardiomyocyte hypertrophy models rescued mitochondrial morphology, membrane potential and ATP generation by restoring peroxisome number and thus reducing oxidative stress (Wang et al. 2023). Furthermore, mice overexpressing the dual peroxisomal/mitochondrial protein TMEM135 (Fig. 1), which negatively regulates peroxisome abundance (Landowski et al. 2023), have a reduced redox ratio in the heart and display mild hypertrophy due to collagen accumulation (Lewis et al. 2018), though the contribution of peroxisomes versus mitochondria to this phenotype is unknown. Conversely, FAR1, which is involved in ether phospholipid synthesis at peroxisomes (Fig. 1), is upregulated in rat cardiomyocytes in response to ER stress induced by ischaemia/reperfusion. FAR1 knockdown reduces cardiomyocyte death in response to ischaemia/reperfusion, suggesting peroxisomal ether phospholipid synthesis negatively regulates cardiomyocyte robustness to stress (Marsh et al. 2021).

### Kidney

Peroxisomes are abundant in cells of the kidney, and especially the epithelial cells of proximal tubules (Grant et al. 2013). Several recent studies have suggested a positive correlation between peroxisome number/function in these cells and improved kidney function under pathophysiological conditions. A proteomics study profiling renal ageing in mice demonstrated a reduction in the expression of peroxisomal proteins, including all enzymes involved in peroxisomal fatty acid oxidation, in the kidneys of old compared to young mice, as well as a reduction in peroxisome number determined by EM (Yi et al. 2020). This is mirrored in mouse models of acute kidney injury, where multiple peroxisomal matrix proteins needed for β-oxidation are also downregulated at the protein level (Burton et al. 2023). Intriguingly, supplementation with nicotinamide mononucleotide, an NAD+ precursor that is thought to reduce age-related organ dysfunction, increased peroxisomal protein expression and peroxisome number in aged mouse kidneys, suggesting that peroxisomes may act to positively support healthy kidney function (Yi et al. 2020). In keeping with this protective hypothesis, knockout mice globally lacking *Sirt5*, encoding a dually targeted mitochondrial/peroxisomal deacetylase, display an increased peroxisome abundance in the proximal tubules and are less sensitive to acute kidney injury. An increase in peroxisomal fatty acid oxidation, both before and after injury, appears to underlie the protective effect of *Sirt5* knockout, since specific inhibition of peroxisomal β-oxidation renders SIRT5-depleted proximal tubule cells as susceptible to in vitro acute kidney injury as controls (Chiba et al. 2019). Consistent with this, *Ehhadh*-knockout mice, which lack the L-bifunctional enzyme that catalyses the second and third steps of peroxisomal β-oxidation, show kidney hypertrophy and signs of proximal tubule injury, though interestingly this is only observed in male mice, suggesting a complex interplay between male-specific hormones and peroxisomal function in the kidney (Ranea-Robles et al. 2021). However, peroxisomal fatty acid oxidation may not counteract kidney dysfunction in all cases—upregulated peroxisomal β-oxidation of dicarboxylic acids in the kidneys of diabetic mice generates the metabolite succinate, which inhibits mitochondrial fatty acid oxidation and causes accumulation of lipids and ROS, in turn exacerbating diabetic nephropathy. Accordingly, specific inhibition of peroxisomal fatty acid oxidation in this model reduces the symptoms and markers of kidney pathology associated with diabetes (Wang et al. 2022b).

In contrast to the evidence above implicating peroxisomes in kidney pathophysiology, a recent study specifically ablating peroxisomes in proximal renal tubules in vivo has suggested they may in fact be dispensable for normal kidney function. Ansermet et al. conditionally knocked out *Pex5* to block peroxisome biogenesis in proximal tubule cells of infant or adult mice, and barring a slight reduction in kidney and body weight in male mice (where the knockout was more complete), observed no changes in kidney morphology or signs of kidney dysfunction (Ansermet et al. 2022). In addition to the predicted changes in peroxisomal pathways in the knockout mice, there was evidence of metabolic reprogramming (e.g., increased mitochondrial fatty acid oxidation) apparently compensating for the loss of peroxisomal functions to maintain kidney homeostasis, altogether suggesting that peroxisomes are not strictly necessary to support healthy renal functions, and also pointing to dysfunction in other systems beyond the kidney as the cause of renal pathologies associated with ZSD (Ansermet et al. 2022).

### Parotid gland

The properties of peroxisomes in parotid glands, which are a major type of salivary gland producing serous saliva, have recently been characterised for the first time. They are highly abundant in all parotid gland cells in both mice and humans, though they vary in number and protein content both within and between cells (Watermann et al. 2021, 2023). A greater number of peroxisomes were observed in striatal duct cells compared to acinar cells, as well as stronger immunofluorescence staining of peroxisomal proteins including catalase, thiolase, and PEX14. Furthermore, mRNAs encoding peroxisomal proteins required for biogenesis, β-oxidation and plasmalogen synthesis, as well as antioxidant enzymes, were expressed throughout parotid glands, suggesting a role for peroxisomal metabolism and oxidative defence in the function of this tissue (Watermann et al. 2021, 2023). Notably, levels of peroxisomal proteins also varied between neoplastic and healthy parotid tissues, with tumour samples exhibiting both up- and downregulation of peroxins and a general upregulation of proteins involved in lipid transport and β-oxidation, indicating a dysregulation of peroxisome biogenesis and lipid metabolism which may be either a driver or consequence of tumour progression (Meyer et al. 2021). Notably, while the trends in peroxisomal mRNA/protein levels varied by tumour type, all neoplastic parotid gland samples expressed higher levels of catalase and other peroxisomal antioxidants, which may be an adaptation to the severe redox imbalance observed in cancers, raising the possibility of targeting the antioxidant activity of peroxisomes as a therapy to reduce tumour cell survival (Meyer et al. 2021).

### Skin

While peroxisomes are well characterised in skin fibroblasts, being a commonly used cell culture model, little attention has been paid to their role in specialised epithelial skin cells such as keratinocytes and melanocytes. Peroxisomes appear to protect melanocytes from light-induced damage, in part through catalase-mediated detoxification of the H_2_O_2_ generated by UV exposure (Maresca et al. 2008). Peroxisomal metabolism also promotes production of melanin, a protective pigment in melanocytes, the abundance of which correlates with catalase levels (Maresca et al. 2008). Supporting this role, recent work has identified a mutation in an enhancer region of the gene encoding GNPAT, a peroxisomal enzyme required for ether phospholipid synthesis, which increases its expression levels in response to UV exposure. This mutation correlates with increased skin pigmentation and tanning ability (melanin production in response to UV exposure) and is more prevalent in Tibetans compared to Han Chinese, representing an adaptation to high levels of UV experienced by this population (Yang et al. 2022). The protective role of peroxisomes in melanocytes also extends to cancer, though via a different mechanism, with increased peroxisomal abundance restricting melanoma growth and metastasis due to the enhanced levels of ROS produced by the concomitant rise in peroxisomal metabolism. ASAH1, an enzyme that hydrolyses ceramides, is significantly overexpressed in melanoma samples. Silencing of ASAH1 suppresses melanoma growth, which can be attributed to the higher levels of intracellular ROS, stemming from increased peroxisome biogenesis (and therefore metabolism) as a result of PPARγ activation by accumulated ceramides (Malvi et al. 2021). However, enhanced peroxisome metabolism in keratinocytes can be detrimental to skin, compromising its barrier function in atopic dermatitis. Homeostasis of VLCFAs is crucial for skin barrier function, as evidenced by lethal dermal dehydration suffered by mice lacking the ER-localised fatty acid elongase ELOVL4 (Li et al. 2007). Accordingly, VLCFA levels are reduced in a mouse model of lesional atopic dermatitis, as a result of elevated peroxisomal β-oxidation via increased ACOX1 expression and activity in keratinocytes, leading to reduced epidermal barrier integrity (Pavel et al. 2021). Altogether, this suggests peroxisomal function contributes to regulating the epidermal lipid signature necessary for healthy skin function.

### Reproductive organs

Both male and female patients with peroxisomal disorders exhibit genital abnormalities, suggesting peroxisomes play an important role in the development and function of the reproductive organs (Wang et al. 2022a). Peroxisomes were initially suspected to play a role in mammalian spermatogenesis as they undergo significant rearrangements in terms of shape, distribution, and protein composition in the germ cells of mouse testes during the formation of mature spermatozoa (Dastig et al. 2011). Several studies have recently confirmed the importance of functional peroxisomes for spermatogenesis in mammals, showing mouse models with testes-specific defects in peroxisomal matrix protein import cannot form mature spermatozoa and are thus infertile (Brauns et al. 2019; Liu et al. 2023). Germ cell specific *Pex5* deletion leads to impaired spermatocyte meiosis and spermatocyte apoptosis (Liu et al. 2023) while a lack of *Pex13* prevents germ cells from undergoing differentiation into spermatids (Brauns et al. 2019). Why import-competent peroxisomes are necessary for healthy spermatogenesis is currently unclear, though the ROS accumulation seen in *Pex5*-deficient germ cells, and the altered lipid profile observed in *Pex13*-deficient testes, may suggest a role for peroxisomal ROS homeostasis and/or lipid metabolism in the process (Brauns et al. 2019; Liu et al. 2023). Consistent with the latter, numerous mouse models lacking enzymes involved in peroxisomal lipid metabolism are also infertile (Wang et al. 2022a).

In contrast to the testes, peroxisomes have been less studied in female reproductive organs. In mouse tissue, PEX14-positive peroxisomes are present in the ovaries and oocytes, being most abundant in the interstitial and granulosa lutein cells which are responsible for the synthesis of steroid hormones and progesterone, respectively (Wang et al. 2022a). Peroxisomes in ovarian tissue are also positive for catalase, with the highest levels observed in the follicular granulosa cells that support the developing oocytes. Given the role of ROS signalling and oxidative stress in folliculogenesis and oocyte maturation, it has been speculated that peroxisomes in the ovary may regulate these processes by controlling ROS homeostasis (Wang et al. 2022a). Furthermore, ovarian cells express the peroxisomal β-oxidation enzyme MFP2, implying that peroxisomal fatty acid metabolism is necessary for normal ovary function, potentially via detoxification of VLCFAs and energy generation to promote the maturation of oocytes within the follicles, as well as being required for steroid biosynthesis in interstitial cells (Wang et al. 2022a). Consistent with this, patients with a defect in MFP2 exhibit ovarian dysgenesis (Pierce et al. 2010). Interestingly, a recently characterised patient with ACBD5 deficiency presents with ovarian insufficiency, which, given the patient’s normal plasma VLCFA levels, could suggest coordinated peroxisome-ER metabolism is also required for healthy ovary function (Rudaks et al. 2023).

### Retina

Almost all patients with a peroxisomal disorder, regardless of severity, present with ocular defects (frequently retinopathy), highlighting the vital roles peroxisomes play in vision, and retinal integrity in particular (Das et al. 2021a). While PEX14/catalase-positive peroxisomes are abundant in all the distinct cell layers that make up the retina, there are significant differences in the expression of peroxisomal proteins between different cell types. For example, catalase, as well as the fatty acid transporters ABCD2 and ABCD3 (Fig. 1), and the active cleaved forms of the peroxisomal β-oxidation enzymes ACAA1 and MFP2, are more highly expressed in mouse retinal pigment epithelium (RPE), compared to the neural retina layers, suggesting peroxisomes in the different retinal cell types may have distinct functions, including in terms of lipid metabolism (Das et al. 2019). Peroxisome-associated lipids, including DHA and very-long-chain PUFAs, are essential components of photoreceptor membranes and are increasingly appreciated to exhibit a strikingly variable distribution both within and between cell membranes (e.g., in rods vs cones) (Agbaga et al. 2018; Sander et al. 2021; Verra et al. 2022). Peroxisomal lipid metabolism can therefore contribute to retinal function by regulating the levels of these metabolites, both in terms of synthesis and, in the case of RPE cells, breaking down potentially toxic VLCFAs liberated by the rapid turnover of photoreceptor outer segments. Accordingly, both patients and mouse models with defects in peroxisomal β-oxidation exhibit ocular phenotypes (Das et al. 2021a). Das et al. recently characterised the retinal phenotypes in a global *Mfp2*-knockout mouse, in which peroxisomal β-oxidation is compromised (Das et al. 2021b). These mice exhibit photoreceptor deterioration leading to decreased visual acuity from 3 weeks of age, suggesting peroxisomal β-oxidation is essential for retinal integrity. Notably, the retinal and plasma lipidome of *Mfp2*-knockout mice at 3 weeks showed a significant reduction in DHA-containing phospholipids, which may underlie the observed retinal dysfunction (Das et al. 2021b).

Despite the clear importance of peroxisomal β-oxidation in maintaining retinal integrity, the function of the peroxisomes in the neural retinal cells themselves remains less clear. In contrast to the early onset retinal degeneration observed in global *Mfp2*-knockout mice (Das et al. 2021b), mice only lacking *Mfp2* or *Pex5* in photoreceptors and bipolar cells display a much milder phenotype, with reduced visual acuity over time but normal retinal morphology and photoreceptor function (Swinkels et al. 2022). Notably, as opposed to the global knockout, DHA-containing lipid levels in the neural retina were normal in retina-specific *Mfp2*-knockout mice, suggesting DHA can be systemically supplied to the retina via peroxisomal synthesis elsewhere, possibly in the liver (Das et al. 2021a). However, VLCFAs were observed to accumulate in the neural retina, along with photoreceptor-bipolar cell synapse loss and retinal inflammation, suggesting a role for local peroxisomal function in these aspects of normal retina function (Swinkels et al. 2022).

In contrast, retinal degeneration is observed in RPE-specific *Mfp2*-knockout mice, seemingly as a secondary effect following RPE dedifferentiation and degeneration. These RPE cells lacking *Mfp2* show accumulations of lipid droplets and very-long-chain PUFAs, resulting from compromised endolysosomal digestion of phagocytosed photoreceptor outer segments (Kocherlakota et al. 2023a). Importantly, inducing degeneration of photoreceptors in global *Mfp2*-knockout mice (by crossing with rd1 mutant mice) rescues the RPE degeneration and reduces lipid accumulation relative to *Mfp2* knockout alone, altogether supporting the idea that the RPE degeneration observed when peroxisomal β-oxidation is compromised stems from the inability of RPE cells to digest the very-long-chain PUFAs coming from the membranes of engulfed photoreceptor outer segments (Kocherlakota et al. 2023a).

Finally, in terms of treating retinal dysfunction associated with loss of peroxisomal functions, injection of wild-type PEX1-encoding AAVs into the retina of the PEX1-Gly844Asp mouse model of mild ZSD has recently been demonstrated to improve ocular phenotypes, showing restoration of peroxisome function in retinal cells alone is sufficient to improve retinal dysfunction, and raising this as an intriguing possible therapy to attenuate the visual symptoms experienced by patients suffering from mild ZSD (Argyriou et al. 2021).

### Pancreas

Peroxisomal metabolism in pancreatic β-cells is increasingly appreciated to be somewhat of a double-edged sword—while necessary for healthy function, it also renders the cells susceptible to lipotoxicity. Mice with a β-cell-specific defect in peroxisomal function, due to knockout of *Pex5* in these cells, have elevated blood glucose levels as a result of reduced glucose-stimulated insulin secretion, suggesting peroxisomes in β-cells are required to maintain glucose homeostasis at an organismal level (Baboota et al. 2019). However, under conditions where (V)LCFAs accumulate, such as in type 2 diabetes mellitus (T2DM), peroxisomal metabolism can contribute to lipotoxicity and β-cell loss (Gehrmann et al. 2010). β-cells contain remarkably low levels of antioxidants, including peroxisomal catalase, meaning that the H_2_O_2_ generated during β-oxidation cannot be efficiently detoxified (Roma and Jonas 2020). Higher levels of VLCFA substrates lead to lipotoxicity in part by increasing peroxisomal β-oxidation rates, generating more H_2_O_2_ which, rather than being decomposed by catalase, can slowly diffuse through the peroxisomal membrane in insulin-producing cells and damage cellular components (Elsner et al. 2011; Laporte et al. 2020). Supporting a role for peroxisomes in driving lipotoxicity, a reduction in peroxisome number in a β-cell line (through PEX11β silencing) decreased palmitate-induced ROS generation and prevented palmitate from reducing glucose-stimulated insulin secretion, thus protecting the cells from lipotoxicity (Blair et al. 2021). In contrast, a complete loss of peroxisome function (through PEX14 silencing) has been shown to exacerbate lipotoxic phenotypes in response to palmitate treatment (Guan et al. 2021), suggesting some level of peroxisomal β-oxidation is required to protect cells from the deleterious effects of accumulated VLCFAs, the most lipotoxic form of free fatty acids to β-cells (Plötz et al. 2019). Furthermore, PEX14 protein expression is significantly downregulated in the foetal pancreas in a mouse model of intrauterine growth restriction, which predisposes animals to developing T2DM later in life (Liu et al. 2019). Beyond β-oxidation, peroxisome-dependent synthesis of ether phospholipids has recently been demonstrated to support β-cell function under conditions where fatty acids are in excess. Short-term inhibition of autophagy in mice rescues high fat diet-induced defects in glucose-stimulated insulin secretion, and augments ether phospholipid synthesis, which is associated with an increased mRNA expression of the peroxisomal chaperone Lonp2 (Chu et al. 2020). Notably, knockdown of the enzyme PexRAP, which catalyses the final peroxisomal step in ether phospholipid synthesis, reduces glucose-stimulated insulin secretion in cultured β-cells, suggesting a role for peroxisomal ether phospholipid metabolism in promoting healthy β-cell function (Chu et al. 2020).

## Mysterious disorders: the loss of peroxisomal functions

The loss of peroxisomal functions has been linked to inherited disorders with often severe and progressive developmental and neurological abnormalities. Peroxisome biogenesis disorders (PBDs) such as ZSD are caused by mutations in PEX genes (Fig. 1, Table 1) encoding for essential peroxisomal biogenesis factors. PEX mutations can impact peroxisomal matrix protein import, membrane assembly or multiplication/proliferation and usually cause the loss of several metabolic functions of peroxisomes. Single enzyme deficiencies (SED) are caused by mutations in peroxisomal enzymes and usually only affect a specific peroxisomal pathway or function. An example is X-linked adrenoleukodystrophy (X-ALD), a progressive neurodegenerative disease with a clinical spectrum that includes primary adrenal insufficiency, progressive myeloneuropathy and cerebral inflammatory disease. X-ALD is the most common peroxisomal disorder (affecting ~ 1:17,000 births), and is caused by mutations in the ABCD1 gene encoding the ALDP protein, an ABC half-transporter in the peroxisomal membrane facilitating the uptake of VLCFA for peroxisomal β-oxidation (Engelen et al. 2012) (Fig. 1). To enable timely therapeutic intervention, X-ALD newborn screening has now been implemented in the United States, Taiwan, and the Netherlands (Barendsen et al. 2020; Albersen et al. 2023).

The physiological, clinical, diagnostic, and treatment aspects of peroxisomal disorders have been covered in recent reviews (Wanders et al. 2017, 2023; Wanders 2018; Cheillan 2020; Honsho et al. 2020b; Steinberg et al. 2020; Bose et al. 2022). Recently discovered new peroxisomal disorders such as deficiencies of ABCD3 (PMP70) (Ferdinandusse et al. 2015), ACBD5 (Yagita et al. 2017; Ferdinandusse et al. 2017; Darwisch et al. 2020) (see “Mysterious shapers, movers, and regulators of peroxisome dynamics”), and ACOX3 (Kim et al. 2020) were also addressed elsewhere (Wanders 2018; Carmichael et al. 2022; Wanders et al. 2023) (Fig. 1).

### Zebrafish and drug screening approaches

Mouse models have been successfully used to study peroxisomal functions and disorders (summarised in Kocherlakota et al. 2023b). Another popular vertebrate model organism for developmental and neurobiology is the zebrafish (*Danio rerio*), due to its high degree of genome conservation with mammals (*D. rerio*). The peroxisomal protein inventory of zebrafish has been recently mapped (Kamoshita et al. 2022). In addition, zebrafish disease models have been developed for X-ALD (Strachan et al. 2017) and for ZSD (Takashima et al. 2021). Similar to X-ALD patients, zebrafish *abcd1* mutants present with impaired development of the central nervous system and the interrenal organ, the zebrafish homolog of the mammalian adrenal cortex. The *abcd1* mutants also display elevated VLCFA levels, cholesterol accumulation, and motor impairments (Strachan et al. 2017; Montoro et al. 2021). The latter results in impaired motor (swimming) function of the *abcd1* mutants, which has been exploited to develop a functional motor behaviour assay for high-throughput small compound screening (Raas et al. 2021). Interestingly, chloroquine was identified as a top hit to rescue the motor behaviour of the zebrafish X-ALD model. ABCD1 deficiency results in an accumulation of saturated VLCFAs. Chloroquine administration increased the expression of stearoyl-CoA desaturase-1 (*scd1*). This enzyme apparently reduced the toxic accumulation of saturated VLCFAs by fatty acid desaturation and metabolic rerouting of fatty acid synthesis towards the generation of less-toxic mono-unsaturated VLCFAs (Raas et al. 2021). Conversely, knockout of *scd1* in zebrafish mimicked the motor phenotype of X-ALD zebrafish. Chloroquine was also able to reduce saturated VLCFAs in human X-ALD fibroblasts and increased SCD1 levels. Treatment of *Abcd1*^*−/y*^ mice with liver X receptor agonists, which increased SCD1 expression, showed VLCFA reductions in X-ALD-relevant tissues. Diminishing VLCFA toxicity by metabolic rerouting of saturated to mono-unsaturated VLCFAs may thus be a therapeutic strategy for X-ALD and other peroxisomal disorders with VLCFA accumulation (Raas et al. 2021).

Small molecule compound screening was also successful in human fibroblasts with a point mutation in PEX1 (PEX1-p.Gly843Asp), which results in a milder ZSD phenotype (Zhang et al. 2010). The point mutation impacts the proper folding of PEX1 and subsequent degradation of the misfolded protein. Compounds with chaperone activity are supposed to improve PEX1 folding and subsequent targeting to peroxisomes, which partially restores peroxisomal matrix protein import and metabolic function (Zhang et al. 2010; Berendse et al. 2013). Those compounds may lead to therapies to promote peroxisome function in patients with a mild peroxisome biogenesis disorder.

### A mysterious role of peroxisomes in ciliopathies

Primary cilia are microtubule-based antenna-like structures at the plasma membrane, which sense and transmit chemical and mechanical signals from outside of the cell. Due to their important roles in tissue development and homeostasis, defects in their formation result in ciliopathies such as polycystic kidney disease, retinal degeneration, polydactyly, and brain malformation (Fliegauf et al. 2007; Goetz and Anderson 2010). Interestingly, evidence has been provided in recent years that peroxisomal dysfunction impairs ciliogenesis. A loss of PEX6 was linked to retinal ciliopathies (Zaki et al. 2016), and knockdown of PEX1 or PEX3 partially suppressed ciliogenesis (Abe et al. 2017). The latter study also showed that Nuclear Dbf2-related kinase 2 (NDR2), which is involved in primary cilium formation, localises to peroxisomes. Furthermore, patients with ZSD often exhibit ciliopathy-related abnormalities. A model has been developed suggesting that peroxisomes deliver cholesterol, which they obtain through lysosome-peroxisome contacts, to the ciliary pocket (Miyamoto et al. 2020; Luo et al. 2021), where it is required for sonic hedgehog signalling, such as in embryogenesis and carcinogenesis. The transport involves microtubules and a PEX14-Rabin8-Rab10-KIFC3 complex. At the base of the primary cilium, PEX14 is thought to mediate an interaction with EHD1/3 on the ciliary pocket membrane. The oxysterol-binding protein ORP3 then facilitates cholesterol transfer from peroxisomes to the ciliary membrane. Experimental support of the model is based on studies of TMEM135 (Fig. 1), which localises to peroxisomes and mediates lysosome-peroxisome contacts. Its depletion, which resulted in an accumulation of cholesterol in lysosomes, also reduced primary ciliogenesis and impaired cholesterol-dependent trafficking and activation of Rab8 (Maharjan et al. 2020). TMEM135 has very recently also been identified as a critical mediator of the peroxisomal regulation of mitochondrial fission and thermogenesis (Beasley et al. 2021; Hu et al. 2023). Furthermore, cells from ZSD patients exhibited decreased levels of ciliary cholesterol and reduced ciliary localization of SMO (smoothened), a cholesterol-activated membrane protein involved in sonic hedgehog signalling (Miyamoto et al. 2020). In line with this, a neomorphic function of tau tubulin kinase 2 (TTBK2), which is associated with spinocerebellar ataxia type 11 (SCA11) has been revealed (Muñoz-Estrada et al. 2023). *TTBK2* variants generate truncated proteins that interfere with primary ciliary trafficking and with sonic hedgehog signalling. SCA11-associated variants contain a PTS1 and reduce peroxisome numbers within the cell and at the base of the cilia, disrupt peroxisome fission, and impair trafficking of ciliary SMO upon sonic hedgehog signalling activation. Overall, these studies highlight a potential link between peroxisome dysfunction, impaired cholesterol transport and ciliopathies.

## Concluding remarks

**Fig. 5:**
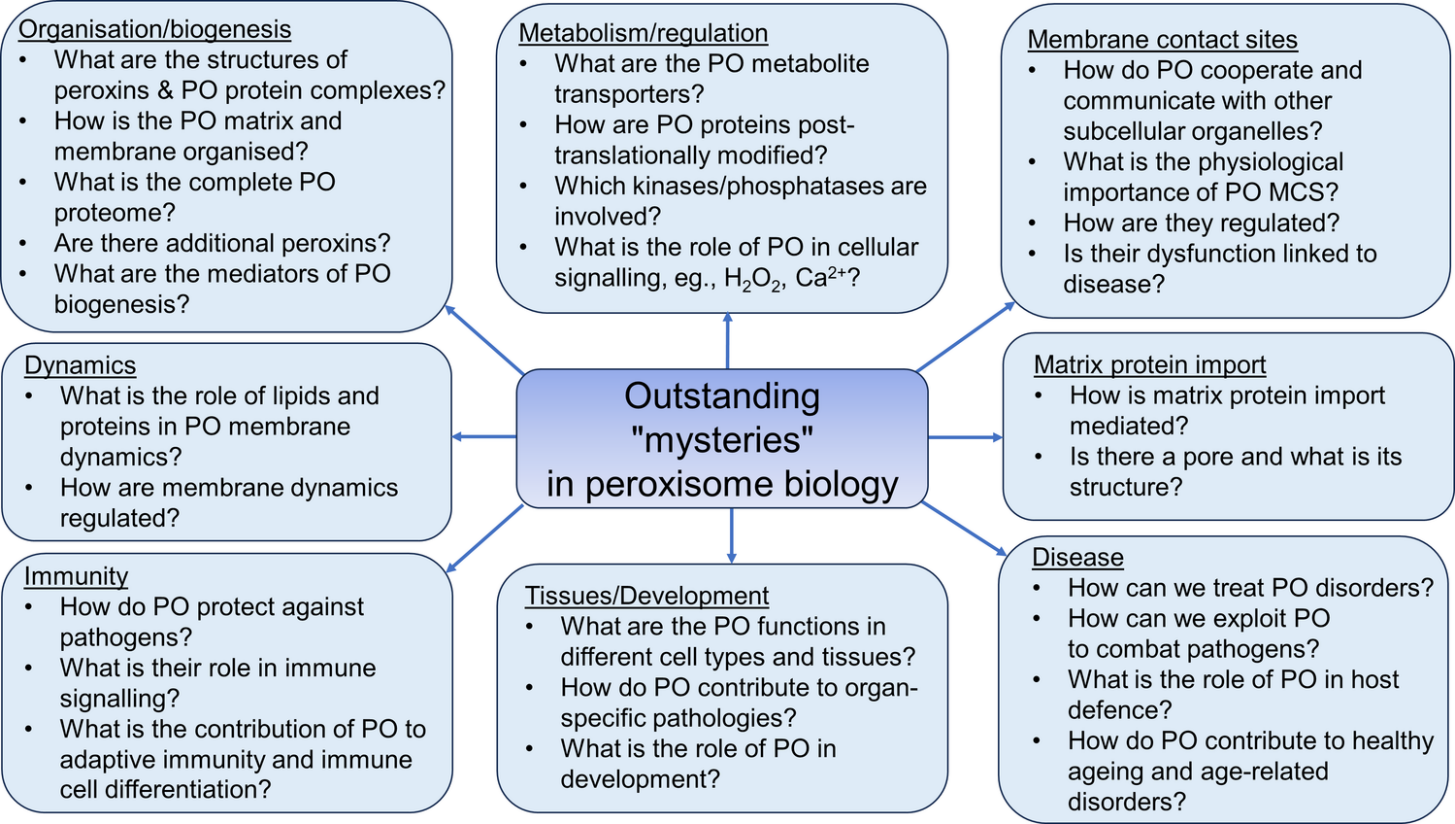
Overview of some “mysteries” of peroxisome biology yet to be solved. For details see text. *PO* peroxisome

Since the publication of our last “mystery” review in 2018 (Islinger et al. 2018), our insights into the metabolic functions of peroxisomes, their biogenesis and removal (Germain and Kim 2020; Li et al. 2021), protein import, and membrane dynamics and division, as well as on peroxisome–organelle membrane contact sites and organelle cooperation, have been further improved. New peroxins have been discovered, and alternative models for the import mechanism of matrix proteins have been proposed, which await further experimental validation (Figs. 1 and 5, Table 1). Progress in cryo-EM and structural biology of peroxisomal proteins has allowed new insights into peroxin structure, peroxisomal protein complexes or disease-relevant transporters (Chen et al. 2022; Jia et al. 2022), and further advancements in this area are expected. With the application of super-resolution microscopy approaches, our current view of peroxisome organisation has improved and will continue to do so. The new roles for peroxisomes in immune and defence mechanisms have opened a new field of peroxisome research and highlight once more how important peroxisomes are for human health and disease, with potential impact on a large number of globally important human diseases. New insights into peroxisome functions in different cell types and tissues have led to a better understanding of their contribution to organ-specific pathologies. Ongoing research focuses on drug screening and gene therapy approaches for the treatment of peroxisomal disorders as well as novel therapies to combat sleeping sickness through glycosome inhibitors (Marciniak et al. 2023) or antiviral therapies. An important and challenging research area is the contribution of peroxisomes to healthy ageing and their role in neuronal plasticity and neurodegenerative disorders (Fig. 5). Future progress here will require a better understanding of the protective roles of peroxisomes, such as their regulation of cellular redox balance, their responses under cellular stress conditions, and their impact on cellular membrane lipids, membrane composition and signalling. Soon, advanced proteomics and CRISPR screens will contribute to the identification of novel mediators of peroxisome biogenesis, additional peroxisome biogenesis factors, and mapping of the complete peroxisomal proteome (Fig. 5). Our knowledge about peroxisome-organelle membrane contacts has profoundly increased in the last years, and a challenge for the future will be to understand the metabolic connections between subcellular organelles, their regulation and physiological importance. It is still not fully understood how peroxisomal functions and abundance are regulated (e.g., through phosphorylation, ubiquitination, SUMOylation), what kinases/phosphatases are involved, or how peroxisomes are linked to cellular signalling pathways and how they act as signalling platforms, for example, through H_2_O_2_ signalling (Lismont et al. 2022) (Fig. 5). There are still many peroxisomal mysteries to be disclosed in the near future, and we are looking forward to providing an update on mysteries 4.0 in a few years’ time.

## Data Availability

The research data supporting this publication are provided within this paper.

## Abbreviations

ACAA1: Acetyl CoA acyltransferase 1/3-ketoacyl-CoA thiolase
ACBD: Acyl-CoA binding domain
ACOX: Acyl-CoA oxidase
DHA: Docosahexaenoic acid
DRP1: Dynamin-related protein 1
EM: Electron microscopy
EPA: Eicosapentaenoic acid
ER: Endoplasmic reticulum
ERAD: ER-associated protein degradation
FFAT: Two phenylalanines in an acidic tract
FIS1: Mitochondrial fission protein 1
IDR: Intrinsically disordered region
IFN: Interferon
LD: Lipid droplet
MAVS: Mitochondrial antiviral signalling protein
MCS: Membrane contact site
MFF: Mitochondrial fission factor
MFP2: Multifunctional protein 2
Mtb: Mycobacterium tuberculosis
PBD: Peroxisome biogenesis disorder
PEX: Peroxin
PMP: Peroxisomal membrane protein
PUFA: Polyunsaturated fatty acid
PPAR: Peroxisome proliferator-activated receptor
PTS: Peroxisomal targeting signal
ROS: Reactive oxygen species
RNS: Reactive nitrogen species
RPE: Retinal pigment epithelium
SMLM: Single-molecule localisation microscopy
STED: Stimulated emission depletion microscopy
T2DM: Type 2 diabetes mellitus
VLCFA: Very-long-chain fatty acid
X-ALD: X-linked adrenoleukodystrophy
ZSD: Zellweger spectrum disorders
ZIKV: Zika virus
